# Reassessing the association between age at menarche and cardiovascular disease: Observational and Mendelian Randomisation analyses

**DOI:** 10.1093/eurjpc/zwaf051

**Published:** 2025-08-05

**Authors:** Lena Tschiderer, Sanne AE Peters, Yvonne T van der Schouw, Stephen Burgess, Janneke Luijken, Cheyenne Bijmolt, Houda Soliman, Adam Butterworth, Angela M Wood, Tammy YN Tong, Christina C Dahm, Lisa Seekircher, Anne Tjønneland, Lene Mellemkjær, Matthias B Schulze, Giovanna Masala, Sabina Sieri, Salvatore Panico, Carlotta Sacerdote, Jolanda MA Boer, WM Monique Verschuren, Carlota Castro-Espin, Dafina Petrova, Sandra M Colorado-Yohar, Conchi Moreno-Iribas, Elisabete Weiderpass, Alicia K Heath, Ioanna Tzoulaki, Peter Willeit, N Charlotte Onland-Moret

**Affiliations:** 1 Institute of Clinical Epidemiology, Public Health, Health Economics, Medical Statistics and Informatics, Medical University of Innsbruck, Innsbruck, Austria; 2 Julius Center for Health Sciences and Primary Care, University Medical Center Utrecht, Utrecht, the Netherlands; 3 The George Institute for Global Health, School of Public Health, Imperial College London, London, United Kingdom; 4 The George Institute for Global Health, University of New South Wales, Sydney, New South Wales, Australia; 5 Department of Public Health and Primary Care, University of Cambridge, Cambridge, United Kingdom; 6 Heart and Lung Research Institute, University of Cambridge, Cambridge, United Kingdom; 7 MRC Biostatistics Unit, School of Clinical Medicine, University of Cambridge, Cambridge, United Kingdom; 8 NIHR Blood and Transplant Research Unit in Donor Health and Behaviour, University of Cambridge, Cambridge, United Kingdom; 9 BHF Centre of Research Excellence, School of Clinical Medicine, Addenbrooke’s Hospital, Cambridge, United Kingdom; 10 Health Data Research UK Cambridge, Wellcome Genome Campus and University of Cambridge, Cambridge, United Kingdom; 11 British Heart Foundation Cardiovascular Epidemiology Unit, Department of Public Health and Primary Care, University of Cambridge, Cambridge, United Kingdom; 12 Victor Phillip Dahdaleh Heart and Lung Research Institute, University of Cambridge, Cambridge, United Kingdom; 13 British Heart Foundation Centre of Research Excellence, University of Cambridge, Cambridge, United Kingdom; 14 Cancer Epidemiology Unit, Nuffield Department of Population Health, University of Oxford, United Kingdom; 15 Department of Public Health, Aarhus University, Aarhus, Denmark; 16 Danish Cancer Institute, Strandboulevarden 49, 2100, Copenhagen, Denmark; 17 Department of Public Health, Section of Environmental Health, Faculty of Health and Medical Sciences, University of Copenhagen, DK-1353 Copenhagen, Denmark; 18 Department of Molecular Epidemiology, German Institute of Human Nutrition Potsdam-Rehbruecke, Nuthetal, Germany; 19 Institute of Nutritional Science, University of Potsdam, Nuthetal, Germany; 20 Clinical Epidemiology Unit, Institute for cancer research, prevention and clinical network (ISPRO), Florence, Italy; 21 Epidemiology and Prevention Unit, Fondazione IRCCS Istituto Nazionale dei Tumori di Milano, Milan, Italy; 22 School of Medicine, Federico II University, Naples, Italy; 23 Unit of Cancer Epidemiology, AOU Città della Salute e della Scienza University Hospital, Turin, Italy; 24 National Institute for Public Health and the Environment, Bilthoven, the Netherlands; 25 Unit of Nutrition and Cancer, Catalan Institute of Oncology-ICO, L’Hospitalet de Llobregat, Barcelona, Spain; 26 Nutrition and Cancer Group, Epidemiology, Public Health, Cancer Prevention and Palliative Care Program, Bellvitge Biomedical Research Institute-IDIBELL, L’Hospitalet de Llobregat, Barcelona, Spain; 27 Escuela Andaluza de Salud Pública (EASP), 18011 Granada, Spain; 28 Instituto de Investigación Biosanitaria ibs.GRANADA, 18012 Granada, Spain; 29 Centro de Investigación Biomédica en Red de Epidemiología y Salud Pública (CIBERESP), 28029 Madrid, Spain; 30 Department of Epidemiology, Murcia Regional Health Council, IMIB-Arrixaca, Murcia, Spain; 31 Research Group on Demography and Health, National Faculty of Public Health, University of Antioquia, Medellín, Colombia; 32 Instituto de Salud Pública y Laboral de Navarra, 31003 Pamplona, Spain; 33 Navarra Institute for Health Research (IdiSNA), 31008 Pamplona, Spain; 34 International Agency for Research on Cancer, World Health Organization, Lyon, France; 35 Department of Epidemiology and Biostatistics, School of Public Health, Imperial College London, London, United Kingdom; 36 Biomedical Research institute, Academy of Athens, Athens, Greece

**Keywords:** age at menarche, cardiovascular risk, individual participant data meta-analysis, Mendelian Randomisation, non-linearity

## Abstract

**Aims:**

Observational studies have shown a U-shaped association between age at menarche (AAM) and cardiovascular disease (CVD). We assessed non-linearity of the observational association and the potential causal relationship between AAM and CVD using data from EPIC-CVD and the UK Biobank.

**Methods:**

We included women without pre-existing myocardial infarction (MI) or stroke at baseline. We estimated hazard ratios for incident MI, ischaemic and haemorrhagic stroke later in life using Cox regression in observational analyses and conducted non-linear Mendelian Randomisation (MR) based on fractional polynomials and linear MR based on inverse-variance weighted regression.

**Results:**

We analysed 283,210 women with a median AAM of 13 (IQR 12-14) years in both EPIC-CVD and the UK Biobank, of which 8,468 experienced MI, 5,501 an ischaemic and 1,887 a haemorrhagic stroke. The association between AAM and MI and ischaemic stroke was U-shaped with higher risks in women aged ≤12 and ≥16 compared to those aged 13 years at menarche. Our MR analyses found no evidence for non-linearity between genetically proxied AAM and any CVD endpoint later in life, but each year higher genetically proxied AAM was related to a lower risk MI (hazard ratio 0.92 [95% CI 0.86-0.99]), but not to ischaemic and haemorrhagic stroke.

**Conclusion:**

This study supported non-linear observational associations between AAM and myocardial infarction and ischaemic stroke. MR analyses suggested a causal relationship between higher AAM and risk of MI without an indication for non-linearity. There was no support for a potential causal link with ischaemic and haemorrhagic stroke.

## Introduction

Average age at menarche (AAM) varies from country to country, ranging from 12 to 16 years,^1^ and a secular decline in age at pubertal onset has been shown worldwide.^2^ Factors that have been related to the onset of menarche include several pre-pubertal anthropometry- and adiposity-related measures such as body fat and body size.^3,4^ In addition, a younger AAM has been linked to several health-related traits including lower lung function, higher risk for psychosocial and mental health problems^5^ and increased risk for breast and endometrial cancer.^6^

Several observational studies have reported an association between AAM and cardiovascular disease (CVD) such as coronary heart disease, stroke, and peripheral artery disease.^4^ In a large-scale individual participant data meta-analysis, both early and late menarche were associated with higher cardiovascular risk suggesting a non-linear U-shaped association.^7^ Moreover, traditional cardiovascular risk factors such as blood pressure and body mass index (BMI) have been suggested to act as potential mediators on the association between AAM and CVD.^8,9^

Multiple Mendelian Randomisation (MR) studies have investigated whether the relationship between AAM and cardiovascular risk factors and CVD events is potentially causal. Younger genetically proxied AAM has been associated with higher fasting blood glucose levels,^10^ higher adult BMI,^11^ and a higher risk of ischaemic heart disease,^12^ coronary artery disease, and heart failure.^9^ Despite the non-linear association seen in observational studies, previous MR studies have assumed a log-linear relationship between genetically proxied AAM and cardiovascular risk. However, it is crucial to know the specific shape of the causal relationship between AAM and CVD and whether both early and late menarche cause CVD later in life. This would enhance our understanding of CVD in women and provide deeper insights into the underlying mechanisms driving the progression of the disease.

We examined whether the shape of association between AAM and CVD risk later in life is non-linear in an observational and a MR analysis setting using data from the European Prospective Investigation into Cancer and Nutrition – Cardiovascular Disease (EPIC-CVD) study and the UK Biobank.

## Methods

Results are presented according to the Strengthening the Reporting of Observational Studies in Epidemiology (STROBE)^13^ and the STROBE-MR Statement.^14^ The STROBE and STROBE-MR checklists are provided in Table S1 and Table S2.

### Study design and data sources

We included data from the EPIC-CVD study and the UK Biobank. Details of these studies have been published previously.^15–17^ The EPIC study recruited more than 500,000 individuals from 23 centres across Europe between 1992 and 2000.^16^ EPIC-CVD is a case-cohort study nested in the EPIC study comprising individuals from a random sub-cohort and all other EPIC participants with incident coronary heart disease and stroke. The UK Biobank is a prospective study conducted in the general population of the UK and included >500,000 participants aged between 40 and 69 years who were recruited between 2006 and 2010.^15^ Women without a history of myocardial infarction (MI) or stroke at baseline were eligible to be included in the present study.

For the MR analysis, we used genetic variants associated with the exposure, i.e., AAM, from a meta-analysis of genome-wide association studies (GWASs).^18^ To obtain genetic associations with the outcome, we used imputed genetic data that passed quality control from EPIC-CVD and the UK Biobank. In EPIC-CVD, genotyping was performed using the Human Core Exome array, Omni Exome Express array, and Illumina 660 Quad array and genotype imputation was based on the Haplotype Reference Consortium.^19^ In the UK Biobank, participants were genotyped with the Affymetrix UK BiLEVE Axiom array and the Affymetrix UKB Axiom Array;^15,20^ genotype imputation was performed using the Haplotype Reference Consortium as well as the UK10K haplotype reference panel.^21^

The GWAS on AAM identified 389 single nucleotide polymorphisms (SNPs).^18^ Of these, we excluded 61 SNPs because no data on these SNPs or any proxy variant were available in EPIC-CVD and nine SNPs because they were palindromic and had a minor allele frequency >0.45 (Figure S1). We harmonised beta coefficients of the SNPs to represent the same effect and non-effect alleles. For EPIC-CVD, we used proxy variants for 21 SNPs that were in high linkage disequilibrium (R^2^>0.8) (Table S3).

AAM was defined as self-reported age at first menstrual periods in both EPIC-CVD and the UK Biobank. In both studies, cardiovascular outcomes were defined using ICD codes. The cardiovascular outcomes studied in this analysis comprised MI (I21-I25 in EPIC-CVD; I21-I23, I24.1, and I25.2 in the UK Biobank), ischaemic stroke (I63-I64), and haemorrhagic stroke (I60-I61). In EPIC-CVD centres, events were ascertained and validated using different methods such as follow-up questionnaires and linkage with morbidity, hospital, and death registries. In the UK Biobank, we used algorithmically defined outcomes (field IDs 42000, 42008, 42010, and 42012) that occurred until September 30, 2021 and were based on hospital admission and death register data. Descriptions of how additional variables were assessed are provided in the Supplementary Methods.

### Statistical analyses

We applied two-sided statistical tests and deemed P-values <0.05 as statistically significant. All analyses were conducted using R 4.0.5 (The R Foundation, Vienna, Austria).

### Descriptive data

We summarised categorical variables as numbers (percentages) and continuous variables as medians (interquartile ranges [IQRs]). For EPIC-CVD, we provide summarised descriptives only in women within the sub-cohort due to the case-cohort design of the study, as those best reflect baseline characteristics of the entire EPIC cohort.

### Observational analysis

In our observational analysis, we multiply imputed missing values in the exposure variable and in the covariates based on chained equations (14 datasets, 30 iterations) using the R-package *mice* v3.14.0.^22^ Details about the imputation process are provided in the Supplementary Methods. We categorised AAM into <12, 12, 13, 14, 15, and ≥16 years. For all analyses, we used women aged 13 years at menarche as the reference group as this was the median AAM in both the EPIC-CVD study and the UK Biobank. We report hazard ratios in each category using quasi variances, which allows head-to-head comparisons of the individual categories of AAM.^23^ We modelled the association between AAM and risk of MI and stroke subtypes using Cox regression analysis. To take the case-cohort design into account, we implemented Prentice-weighted Cox regression for EPIC-CVD.^24^ Follow-up time was defined as time to first MI, ischaemic stroke, or haemorrhagic stroke, death, or loss to follow-up, whichever occurred first. Consequently, if a person experienced more than one CVD event, e.g. both a MI and an ischaemic stroke, only the first event would contribute to any of the Cox regression analyses. We obtained hazard ratios in each study separately and combined them using multivariate random-effects meta-analysis.^25^ Moreover, we adjusted for age, education (high, medium versus low), smoking status (current, ex versus never), and BMI (kg/m^2^). For EPIC-CVD, we additionally stratified by country.

In a sensitivity analysis, we restricted our primary observational analyses to women who were included in the MR study to enhance comparability.

### MR study

In the MR study, we included all women with (1) genetic data on the SNPs used in our instrumental variable that passed quality control and (2) data on AAM. We calculated F-statistics and R^2^ for each study by regressing the genetic instruments on AAM using women from the random sub-cohort for EPIC-CVD.

To study the distribution of genetically proxied AAM across several participant characteristics, we calculated a genetic risk score (GRS) based on the GWAS summary-level data on genetic associations with AAM.^18^ This GRS contained all variants included in the instrumental variable used in the MR analysis weighted by the corresponding effect size obtained from the GWAS on AAM.^18^ We then compared thirds of the GRS across the variables age, hypertension, diabetes mellitus, BMI, height, smoking status, education, total cholesterol, C-reactive protein, use of oral contraceptive pills, menopausal status, and age at menopause. For EPIC-CVD, we included sub-cohort participants only to enhance comparability.

We applied non-linear MR analysis based on fractional polynomials^26^ using the R-package *SUMnlmr*^27^ based on the doubly-ranked stratification method as suggested by Tian et al., which ranks individuals into pre-strata based on their level of the GRS, before stratifying individuals by ranking them within each pre-stratum according to their phenotypic AAM.^28^ The number of pre-strata depends on the sample size of the study and the number of strata selected (sample size = number of strata × number of pre-strata).^28^ For the present analysis, we selected ten strata. In each of the strata, we conducted Cox regression analysis in the UK Biobank and Prentice-weighted Cox regression analysis in EPIC-CVD^24^ to obtain genetic associations with MI, and ischaemic and haemorrhagic stroke based on the GRS. We used age as the underlying time scale. Moreover, in each of the strata, we performed linear regression analysis regressing AAM on the GRS to obtain genetic associations with AAM. In EPIC-CVD, we adjusted for age, genotype array, and the first 10 genetic principal components and stratified by country. In the UK Biobank, we adjusted for age, genotype array, and the first 16 genetic principal components. Study-specific estimates in each stratum were combined using fixed-effect meta-analysis. We used fixed-effect meta-analysis because we assumed that the studies would estimate a common true effect size. Fractional polynomials were fitted using the *frac_poly_summ_mr* function from the R-package *SUMnlmr*.^27^ Non-linearity was assessed by a fractional polynomial non-linearity test comparing a non-linear fractional polynomial model with a linear model.^26,27^ In order to study selection bias potentially introduced by stratification, we assessed the relationship of the strata on AAM with several other traits as suggested previously.^29^

For situations where no evidence of non-linearity was found, we also report results of linear MR (using the R-package *MendelianRandomization* v0.6.0^30^) performing inverse-variance weighted regression as main analysis and simple and weighted median regression, MR-Egger, and MR-PRESSO^31^ as sensitivity analyses. To obtain genetic associations with MI, ischaemic and haemorrhagic stroke, we used the same methods as described above, i.e., we performed Prentice-weighted Cox regression in EPIC-CVD^24^ and Cox regression analysis in the UK Biobank using age as the underlying time scale adjusting (and stratifying) for the same set of variables and combining study-specific hazard ratios using fixed-effect meta-analysis.

## Results

### Participants

**Figure 1:**
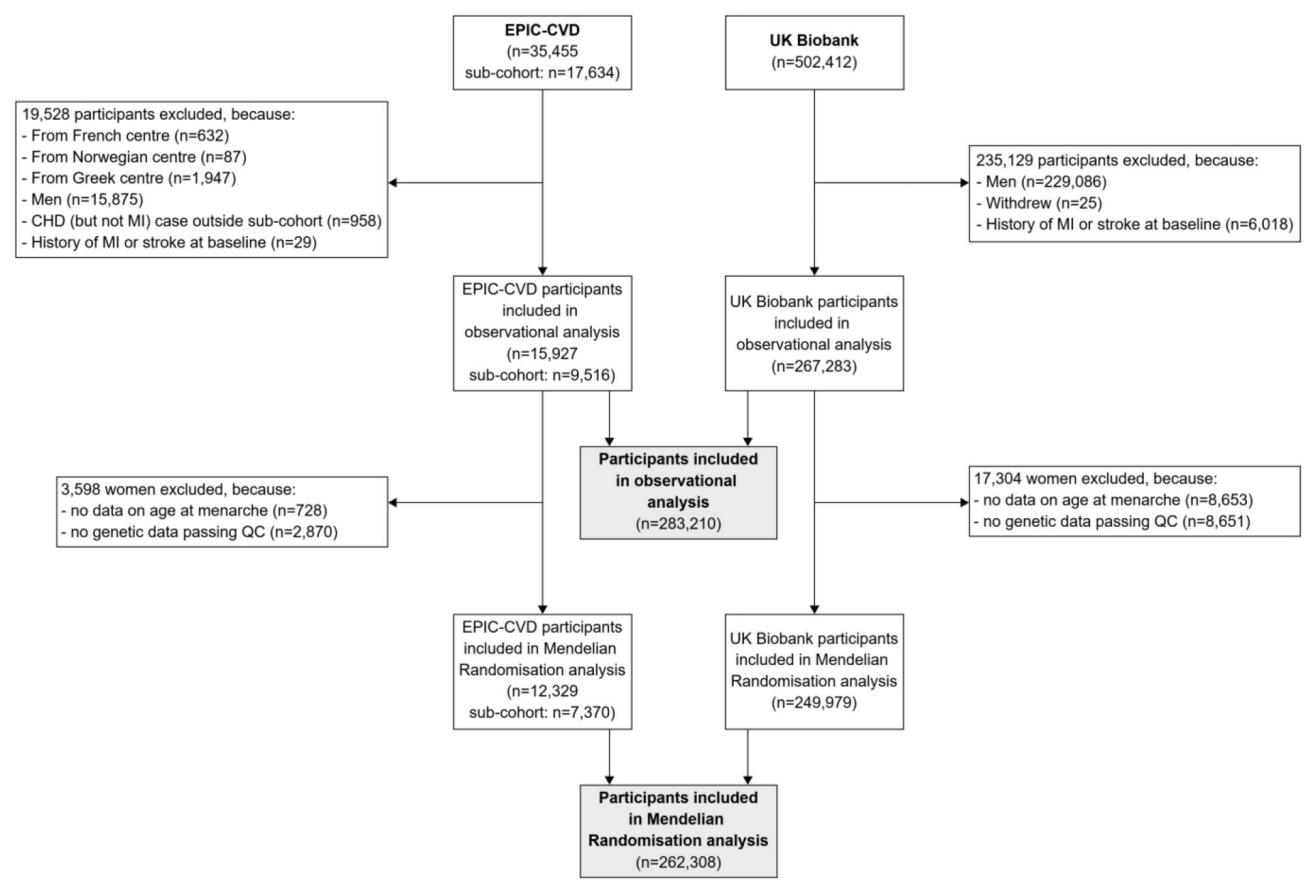
Flow diagram Abbreviations: CHD, coronary heart disease; EPIC-CVD, European Prospective Investigation into Cancer and Nutrition – Cardiovascular Disease; MI, myocardial infarction; QC, quality control.

The study flow diagram is shown in Figure 1 and numbers of participants excluded are described in detail in the Supplementary methods. 283,210 women (15,927 from EPIC-CVD [9,516 from the EPIC-CVD sub-cohort], 267,283 from the UK Biobank) were included in the observational analysis and 262,308 women (12,329 from EPIC-CVD [7,370 from the EPIC-CVD sub-cohort], 249,979 from the UK Biobank) were included in the MR analysis.

### Descriptive data

An overview of the participant characteristics is provided in Table 1. Median baseline age was 52.1 years (IQR 45.5-59.0) and 57.0 years (IQR 50.0-63.0) in the EPIC-CVD sub-cohort and the UK Biobank, respectively. The median AAM was 13.0 years (IQR 12.0-14.0) in both the EPIC-CVD sub-cohort and in the UK Biobank.

Median time to event or end of follow-up was 12.9 years (IQR 10.8-14.3) in the EPIC-CVD sub-cohort and 12.6 years (IQR 11.8-13.3) in the UK Biobank. 8,468 women experienced a MI (3,736 in EPIC-CVD [of whom 184 in the EPIC-CVD sub-cohort], 4,732 in the UK Biobank), 5,501 women experienced an ischaemic stroke (2,542 in EPIC-CVD [of whom 137 in the EPIC-CVD sub-cohort], 2,959 in the UK Biobank), and 1,887 women experienced haemorrhagic stroke (725 in EPIC-CVD [of whom 44 in the EPIC-CVD sub-cohort], 1,162 in the UK Biobank).

### Observational analysis

**Figure 2:**
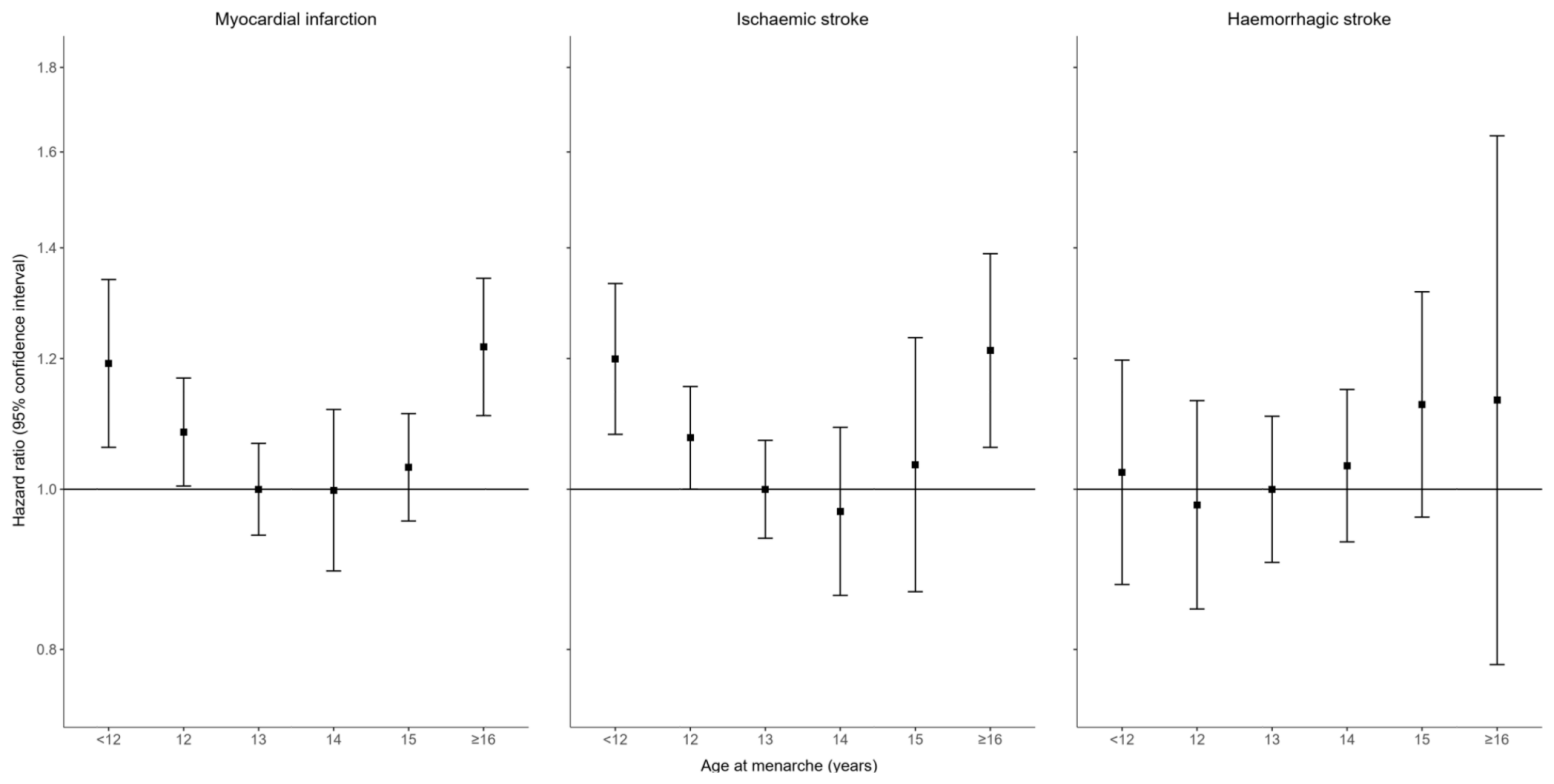
Observational results for the associations between age at menarche and risks of myocardial infarction and ischaemic and haemorrhagic stroke. The models were adjusted for age, education (high, medium versus low), smoking status (current, ex versus never), and body mass index (kg/m^2^). For EPIC-CVD, models were stratified by country. Results are presented based on quasi variances using 13 years at menarche as reference category.

Results of our observational analysis are provided in Figure 2 and Table S4. The associations between AAM and risks of MI and ischaemic stroke were U-shaped, with higher risks for women aged ≤12 years or ≥16 years at menarche as compared to those aged 13 years. We found no significant association between AAM and risk of haemorrhagic stroke. As depicted in Table S5 results remained largely robust when restricting the observational analysis to women who were included in the MR analysis.

### MR analysis

SNP-specific genetic associations with cardiovascular outcomes are provided in Table S3. The R^2^ of our genetic instrument was 10.8% and 5.6% in EPIC-CVD and the UK Biobank, respectively. The F-statistic was 2.67 in EPIC-CVD and 46.00 in the UK Biobank. As depicted in Table S6, the participant characteristics were largely similar across thirds of the GRS.

**Figure 3:**
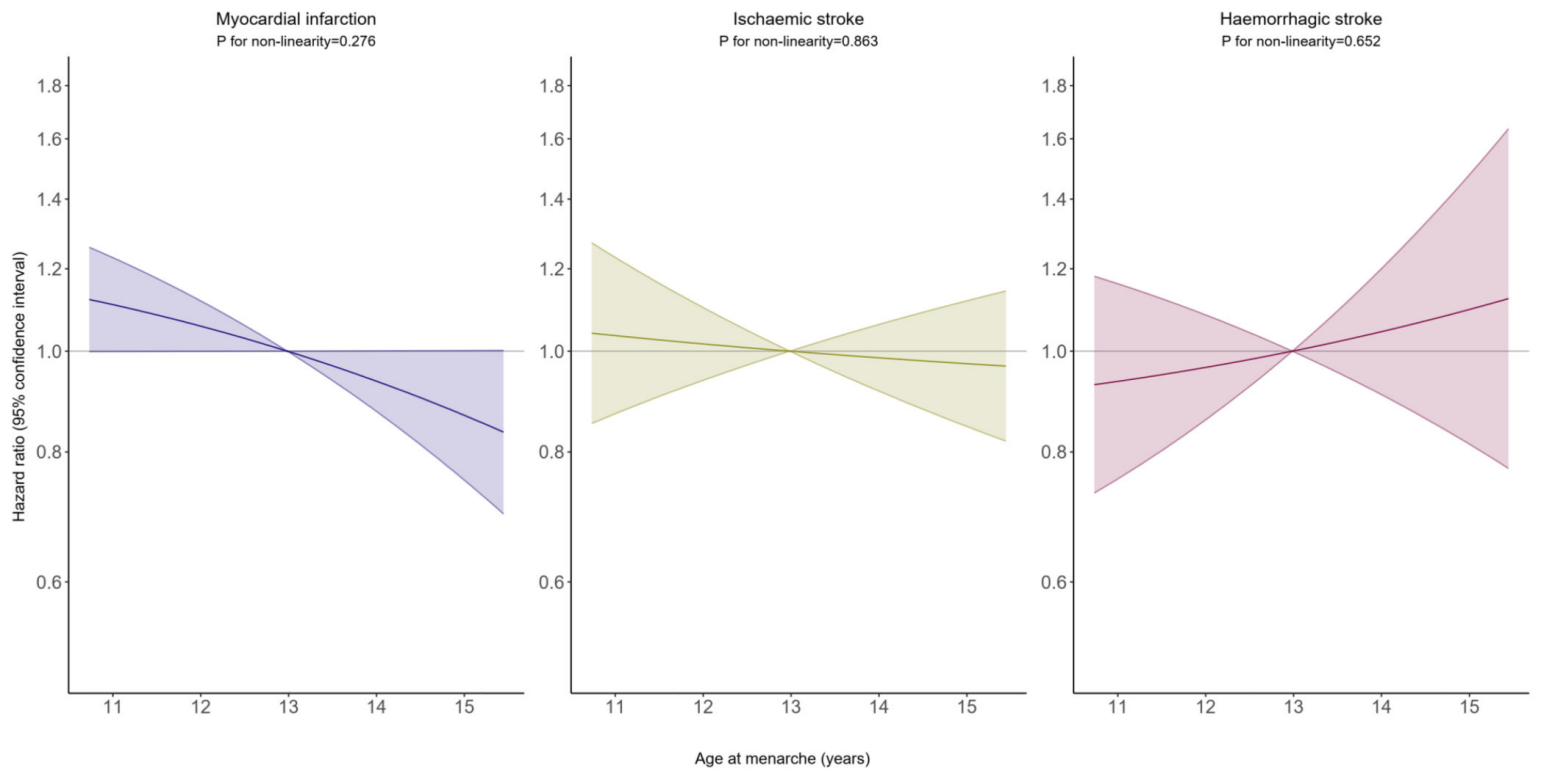
Mendelian Randomisation analysis for the relationships between age at menarche and risks of myocardial infarction and ischaemic and haemorrhagic stroke based on fractional polynomials. The P-value for non-linearity is based on a fractional polynomial non-linearity test comparing a non-linear fractional polynomial model with a linear model.^26,27^ For EPIC-CVD, models were adjusted for age, genotyping array, and the first 10 genetic principal components, and stratified by country. For the UK Biobank, models were adjusted for age, genotyping array, and the first 16 genetic principal components.

Figure 3 shows the results of the non-linear MR analysis. We found no evidence for statistically significant deviation from linearity in the associations between genetically proxied AAM and MI, ischaemic and haemorrhagic stroke (all P-values for non-linearity >0.05). When analysing the relationship of genetically proxied AAM and other traits across strata of AAM, we found no specific patterns of association across the traits (Figure S2). In addition, there was no clear evidence for a relationship with other traits, except for BMI, for which we found inverse associations across all strata of AAM, and for height, for which we found a positive association across all strata.

When conducting linear MR analysis using inverse-variance weighted regression (see Figure S3, and Figure S4 and Figure S5 for study-specific results), a 1-year higher genetically proxied AAM was related to a lower risk of MI with a hazard ratio of 0.92 (95% CI 0.86-0.99; P=0.036). No significant associations were found between genetically proxied AAM and risks of ischaemic and haemorrhagic stroke, with hazard ratios of 1.00 (0.91-1.09; P=0.929) and 1.02 (0.89-1.17; P=0.805), respectively. Findings were consistent when applying simple and weighted median regression although they did not reach statistical significance for MI and MR-Egger suggested no evidence for directional pleiotropy. MR-PRESSO detected only one significant outlier when studying the relationship between genetically proxied AAM and MI in EPIC-CVD. However, when excluding this SNP from the analysis, the result was almost identical. MR-PRESSO detected no statistically significant outliers for any other analysis.

## Discussion

In this study, we found U-shaped associations between AAM and risks of MI and ischaemic stroke later in life based on observational data, but no significant association with haemorrhagic stroke. This non-linear shape of association was not confirmed by MR analysis. However, we found a statistically significant relation per year younger genetically proxied AAM with higher risk of MI but not with ischaemic or haemorrhagic stroke.

### Comparison to findings from the literature

A previous large-scale individual-participant data meta-analysis including data on more than 300,000 participants from twelve studies (including data from the UK Biobank) reported similar results to our observational analysis.^7^ In that meta-analysis, the association between AAM and risk of CVD was U-shaped. Compared to women aged 13 at menarche, those aged ≤10, 11, and 16 years at menarche had a statistically significantly higher risk for CVD with hazard ratios of 1.16 (95% CI 1.04-1.29), 1.15 (1.07-1.22), and 1.15 (1.06-1.24), respectively.^7^ Shapes of associations were also U-shaped for coronary heart disease and stroke.^7^

To the best of our knowledge this is the first MR study that investigated the specific shape of association between genetically proxied AAM and risk of CVD later in life as previously conducted MR studies assumed a linear association. In the present analysis, we found no evidence for deviation from a linear relationship between genetically proxied AAM and risk of any CVD endpoint. Consequently, the linearity assumption of previous MR studies seems to be valid. These studies also reported similar findings to those herein. A MR analysis based on summary-level data reported significant associations between genetic liability to older AAM and lower risk of coronary artery disease with an odds ratio of 0.91 (95% CI 0.88-0.94) per year older genetically proxied AAM, but no statistically significant relationship with the risks of ischaemic stroke and total stroke.^9^ Findings were similar in a sensitivity analysis restricted to women from the UK Biobank for genetic association with outcomes, with an odds ratio for coronary artery disease of 0.91 (0.85-0.96) per year older genetically proxied AAM.^9^ Another summary-level MR analysis reported older genetically proxied AAM to be related to a lower risk of ischaemic heart disease with an odds ratio of 0.80 (0.72-0.88).^12^ In an additional linear MR analysis, we also corroborate previous findings by reporting a hazard ratio for MI of 0.92 (0.86-0.99) per year older genetically proxied AAM and no statistically significant relationships with ischaemic stroke. We did not find significant relationships with haemorrhagic stroke. Contrarily, another summary-level MR study reported a potential causal relationship between older genetically proxied AAM and lower risk of intracerebral haemorrhage.^32^

### Later menarche and risk of MI

Our observational analysis revealed a U-shaped association between AAM and risk of MI. The corresponding MR analysis suggested no non-linearity but a significant relationship with higher risk of MI per year younger genetically proxied AAM. This raises the question of why women with later AAM are at higher risk for MI in observational studies and whether observational findings are affected by confounding. Several pre-pubertal adiposity measures have been suggested to be causally related to later AAM, including lower BMI, lower total body fat, and lower waist-height ratio.^3^ A GWAS on childhood body fatness also reported a genetic correlation between AAM and childhood body fatness.^33^ In addition, smaller early life body size (self-reported variable, asking whether individuals considered themselves as being thinner, plumper, or average at age ten) and lower childhood BMI have been causally related to a lower risk of MI and coronary artery disease, respectively.^34,35^ Potential additional confounding factors in the observational analyses are childhood undernutrition and anorexia nervosa, which have been related to both late menarche^36,37^ as well as to cardiovascular complications.^38,39^ A systematic review on non-genetic determinants of AAM additionally highlighted potential implications of psychological factors, such as adverse childhood experiences and stressful family situations, and environmental factors on the timing of menarche.^40^ Although there are several theories on causes of later menarche, the specific mechanisms leading to higher observational risk of MI in women with late menarche still have to be elucidated. Future studies that investigate shared risk factors for both later menarche and risk of MI are needed to better understand the potential driving factors for the non-linear observational association between age at menarche and risk of MI.

### Implications

According to our study, women with earlier menarche are at higher risk for MI and ischaemic stroke and earlier menarche appears to be causally related to MI. Several factors have been proposed to lie on the causal pathway between AAM and MI. Adult BMI has been suggested as a main driver of higher risk of coronary artery disease in women with lower genetically proxied AAM.^9,41^ In addition, type 2 diabetes mellitus (to which BMI is also causally linked^42^) has been proposed as a mediator on the effect of genetically proxied AAM on risk of coronary artery disease.^9^ Another MR mediation analysis suggested systolic blood pressure as a main mediator of the effect of genetically proxied AAM on coronary artery disease with a proportion mediated of 29%.^43^ In addition, a recent MR analysis found that 37.5% of the relationship between AAM and MI was mediated by genetically proxied current smoking behaviour, and levels of glycated haemoglobin, systolic blood pressure, and triglycerides.^8^ These results suggest that early menarche may cause cardiometabolic alterations on various levels, ultimately potentially leading to MI. However, the majority of these alterations may be traced back to obesity-related factors as that the relationship between AAM and hypertension^44,45^ and diabetes mellitus^46^ is also (partly) mediated by BMI. The reason for the causal association being specific to MI – but not ischaemic stroke – needs to be clarified. Prior MR studies have suggested a minor role of adiposity in the development of stroke.^47^ As BMI may be one of the main drivers of the association between genetically proxied AAM and risk of MI, this could be a potential reason for the apparent lack of causal relationship with stroke risk. However, it is clear that cardiometabolic risk profiles of women with early menarche require specific attention. Raising awareness of elevated cardiovascular risk and implementing strategies to reduce the risk to develop clinically manifest disease, such as MI, is, therefore, important to provide the opportunity for a long-term healthy life in women.

### Strengths and limitations

Our study has several strengths. It comprises meta-analyses of both observational and MR analyses. In addition, it is the first MR analysis on AAM considering a non-linear shape of association. Moreover, our genetic instrument is based on findings from a large-scale GWAS that meta-analysed data from multiple studies.^18^ Furthermore, we used individual-level data from EPIC-CVD and the UK Biobank, which allowed us to restrict the data to women when obtaining genetic associations with cardiovascular outcomes rather than using sex-combined effect estimates. In addition, there have been criticisms of non-linear MR that can introduce bias.^29^ Therefore, we applied the doubly-ranked method, which has been shown to produce more robust results.^28,29^ Moreover, we have conducted a sensitivity analysis investigating the association between genetically proxied AAM and various traits across strata, which revealed no specific shape of association. Our study also has limitations. Both our exposure and outcome data were based on women who are mainly of European ancestry limiting the generalisability of our findings to women of other ancestries. Consequently, additional studies are needed in order to study the specific shape of association between AAM and CVD risk in populations of non-European ancestry. Previous observational studies indicated that associations between AAM and CVD risk in populations of Asian ancestry could be different from populations of European ancestry. For instance, a large-scale study conducted in Korea including >1 million postmenopausal women suggested a linear association between AAM and risk of MI and later menarche was linked to a higher risk of MI.^48^ Similarly, in an analysis of >1 million pre-menopausal Korean women, later menarche was related to a higher risk of CVD.^49^ Moreover, a systematic review highlighted that previous studies in individuals of Asian ancestry reported conflicting results.^4^ In addition, age at pubertal onset has been shown to decline secularly.^2^ Therefore, we cannot draw any conclusions about women born in decades other than those included in EPIC-CVD and the UK Biobank. Moreover, AAM was self-reported and may consequently be prone to recall bias, especially, given that AAM is an early life exposure. However, an analysis within the Newton Girls Study demonstrated a significantly high correlation between original and recalled AAM after 33 years.^50^ Furthermore, data on pre-pubertal cardiovascular risk factors were not available. Consequently, we could not account for these factors in our observational analyses. Another limitation is that the GWAS we used for genetic associations with AAM included data from EPIC and the UK Biobank preventing us from performing two-sample MR analysis with non-overlapping samples. In addition, as EPIC-CVD is a case cohort study on coronary heart disease and stroke events by design, it was not possible to analyse additional cardiovascular endpoints such as, for instance, heart failure and peripheral artery disease. Further studies are needed in order to investigate the shape of association between AAM and risk of additional cardiovascular outcomes. Moreover, although we aimed to harmonise endpoint definitions, the definition for MI differed slightly between the studies. When conducting MR analysis, there are three core assumptions on the genetic instrument: (1) it is associated with AAM, (2) it is not associated with AAM via confounding pathways, and (3) it can influence cardiovascular risk only via AAM. We checked whether our genetic instrument is likely to fulfil these assumptions. To fulfil the first assumption and select SNPs for our instrumental variable that are related to AAM, we used data from a large-scale GWAS on AAM.^18^ In addition, we assessed the association of our instrumental variables with phenotypic AAM. While in the UK Biobank the F-statistic was relatively high with 46.00, it was only 2.67 in EPIC-CVD due to the lower sample size. This could have resulted in weak instrument bias in EPIC-CVD. As the samples of the genetic associations with the exposure and outcome were overlapping, weak instrument bias could bias the results towards the observational association. However, R^2^ was 5.6% in the UK Biobank and was even higher with 10.8% in EPIC-CVD. The second assumption is usually very likely to hold as genetic variants are determined at conception. This assumption could be violated by population stratification. However, our MR analysis was mainly based on individuals of European ancestry and we additional adjusted our analyses for genetic principal components. It can usually not be excluded that the third assumption is violated in MR analysis. Nevertheless, we have conducted a range of sensitivity analysis in order to investigate the violation of this assumption. We studied the distribution of participant characteristics across the GRS for AAM and found that participant characteristics were distributed homogeneously across thirds of the GRS for AAM, which indicates that our instrumental variable is not related to these potential confounding factors. Moreover, MR-Egger identified no direct pleiotropy (all P-values of intercepts >0.05) and no significant outliers were detected by MR-PRESSO, suggesting that our findings are unlikely to be driven by pleiotropic effects.

## Conclusions

In this large European study, we found U-shaped observational associations between AAM and risks of MI and ischaemic stroke later in life. MR analysis suggested no non-linear relationship between genetically proxied AAM and any cardiovascular outcome later in life, yet each year of higher genetically proxied AAM was related to a lower risk of MI.

## Supplementary Material

Supplementary Material

## Figures and Tables

**Table 1:** Baseline characteristics.

	EPIC-CVD^a^ (n=9,516)		UK Biobank (n=267,283)	
Characteristic	n^b^	Median [IQR], n (%)	n^b^	Median [IQR], n (%)
Age, years	9,516	52.1 [45.5-59.0]	267,282	57.0 [50.0-63.0]
Age at menarche, years	9,081	13.0 [12.0-14.0]	258,630	13.0 [12.0-14.0]
Hypertension	9,445	3,239 (34.3%)	266,172	116,493 (43.8%)
Diabetes mellitus	8,779	227 (2.6%)	266,121	9,609 (3.6%)
Body mass index, kg/m^2^	9,447	25.1 [22.7-28.4]	265,907	26.1 [23.4-29.7]
Height, cm	9,481	161.2 [156.8-166.0]	266,189	162.0 [158.0-167.0]
Smoking status	9,439		265,833	
Never		5,267 (55.8%)		159,230 (59.9%)
Ex		1,990 (21.1%)		83,107 (31.3%)
Current		2,182 (23.1%)		23,496 (8.8%)
Education	9,349		262,098	
Low		4,135 (44.2%)		43,977 (16.8%)
Medium		1,353 (14.5%)		66,861 (25.5%)
High		3,861 (41.3%)		151,260 (57.7%)
Total cholesterol, mmol/L	9,138	5.8 [5.1-6.6]	249,012	5.8 [5.1-6.6]
C-reactive protein, mg/L	9,134	1.1 [0.5-2.5]	248,590	1.4 [0.6-3.0]
Ever use of OCP	9,154	5,067 (55.4%)	265,913	215,982 (81.2%)
Postmenopausal	9,516	5,292 (55.6%)	267,282	194,224 (72.7%)
Age at menopause, years	4,754	49.0 [45.0-52.0]	182,351	50.0 [45.0-52.0]

^a^Including data from the EPIC-CVD sub-cohort, ^b^Number of non-missing values. Abbreviations: EPIC-CVD, European Prospective Investigation into Cancer and Nutrition – Cardiovascular Disease; IQR, interquartile range; OCP, oral contraceptive pill.

## Data Availability

The data underlying this article can be requested via the UK Biobank website (https://www.ukbiobank.ac.uk/enable-your-research/apply-for-access).

## Lay summary

This study reassessed the relationship between age at menarche and risk of cardiovascular disease based on observational analyses and Mendelian Randomisation studies.

- In observational analyses, the association between age at menarche and myocardial infarction and ischaemic stroke was U-shaped, which was not confirmed by Mendelian Randomisation studies.
- This study contributes valuable insights into the shape of association between age at menarche and risk of cardiovascular disease, which ultimately leads to an improved understanding of the development of cardiovascular disease in women.
