# Supplementary material for: Reassessing the association between age at menarche and cardiovascular disease: Observational and Mendelian Randomisation analyses
Source: Eur J Prev Cardiol. Author manuscript; Available in PMC 2025 Aug 15. (PMC7618008; doi:10.1093/eurjpc/zwaf051)
Supplement: Supplementary Material [file EMS207595-supplement-Supplementary_Material.docx]

Supplementary Material

**Reassessing the association between age at menarche and cardiovascular disease: Observational and Mendelian Randomisation analyses**

# Supplementary methods

## Participants eligible for inclusion

Of the 35,455 EPIC-CVD participants, we excluded 87 from Norway because data on essential covariates were unavailable, 632 from France because no follow-up for stroke was available, 1,947 from Greece because of data sharing restrictions, 15,875 because they were men, 958 because they were coronary heart disease (but not myocardial infarction) cases outside the sub-cohort, and 29 because they had a history of myocardial infarction or stroke at baseline Of the 502,412 participants from the UK Biobank, we excluded 229,086 because they were men, 25 because they withdrew from the study, and 6,018 because they had a history of myocardial infarction or stroke at study baseline. For the Mendelian Randomisation analysis, another 9,381 (728 from EPIC-CVD, 8,653 from the UK Biobank) participants were excluded because they had no information on age at menarche and 11,521 (2,870 from EPIC-CVD, 8,651 from the UK Biobank) because of missing genetic data passing the quality control checks.

## Assessment of additional variables

In the UK Biobank, systolic and diastolic blood pressure were measured based on automated reading and the average of two blood pressure measures taken a few moments apart were used in the analyses. In both studies, women were considered hypertensive if they had a systolic blood pressure >140 mmHg, a diastolic blood pressure >90 mmHg, or reported use of antihypertensive medication. In EPIC-CVD, women with a self-reported history of hypertension were also defined as hypertensive. History of diabetes mellitus was self-reported in both studies. Body mass index was calculated by dividing weight (in kg) by height (in m^2^). Smoking status refers to tobacco smoking and was divided into never, ex, and current smoking. Total cholesterol, high-density lipoprotein cholesterol, triglycerides, apolipoprotein A1, apolipoprotein B, and high-sensitivity C-reactive protein were measured in serum samples at baseline on a Roche auto-analyser at Stichting Huisartsen Laboratorium (Etten-Leur, The Netherlands) in EPIC-CVD and using a Beckman Coulter AU5800 platform in UK Biobank. In the UK Biobank, physical activity was defined according to the International Physical Activity Questionnaire (IPAQ) scoring protocol (short forms).^51^ In EPIC-CVD, physical activity was defined using the Cambridge physical activity index.^52^ Education was defined according to highest level of education completed and was categorised into low, medium, and high. In EPIC-CVD, low education was defined as no schooling or primary school, medium as secondary school, and high as vocational school or university. In the UK Biobank, low education was defined as no schooling, medium as A levels/AS levels or equivalent, O levels/General Certificate of Secondary Educations or equivalent, or Certificate of Secondary Educations or equivalent, and high as National Vocational Qualification, Higher National Certificates, Higher National Diplomas, or equivalent, or other professional qualifications. In the UK Biobank, Townsend deprivation index was used to measure socioeconomic status. Women were considered postmenopausal if they (1) experienced natural menopause (defined as stopping of periods in UK Biobank and as reporting no menses for one year or longer due to natural menopause in EPIC-CVD), (2) had had a unilateral or bilateral ovariectomy in EPIC-CVD or bilateral ovariectomy in UK Biobank, or (3) had had a hysterectomy. In case no menopause information was provided, women aged >54 years were considered postmenopausal. Age at menopause was defined as age of a woman’s last menstruation, or age at ovariectomy or hysterectomy.

## Multiple imputation of missing values

We conducted multiple imputation under the assumption that variables are missing at random and included a wide range of variables in our imputation models to minimise the possibility of missing data being missing not at random. In both studies, the imputation process included the variables age, total cholesterol, high-density lipoprotein cholesterol, triglycerides, apolipoprotein A1, apolipoprotein B, log-transformed high-sensitivity C-reactive protein, body mass index, physical activity, height, history of diabetes mellitus, history of hypertension, smoking status, education, menopausal status, age at menopause, ever use of oral contraceptive pill, age at menarche, the cardiovascular outcome statuses, time to first cardiovascular disease event, and the Nelson-Aalen estimator (weighted with the sample fraction for EPIC-CVD). In EPIC-CVD the variables centre and country were additionally included. For the UK Biobank, we also used systolic and diastolic blood pressure, and Townsend deprivation index. All variables that contained missing values (i.e., all except age, menopausal status, cardiovascular outcome statuses, time to first cardiovascular disease event, and the Nelson-Aalen estimator and centre and country for EPIC-CVD) were imputed. We included age at menarche as a continuous variable in the imputation process. In addition, we used passive multiple imputation for categories of age at menarche. In case two predictor variables were strongly correlated, i.e., their Pearson correlation coefficient was >0.7 or <-0.7, we only used the predictor variable for multiple imputation that had the strongest correlation with the variable to be imputed. We used the following imputation methods: predictive mean matching for continuous variables, logistic regression for binary variables, and polytomous regression for categorical variables with more than two categories. We checked the convergence of the imputation algorithm by visually inspecting convergence plots. We used within-study multiple imputation, imputing data in each study separately, combining the results by Rubin’s rule, and meta-analysing them across the studies.^53^

# Supplementary tables

Table S1. STROBE checklist.

|  | **Item No.** | **Recommendation** | **Page No.** |
| --- | --- | --- | --- |
| **Title and abstract** | 1 | (*a*) Indicate the study’s design with a commonly used term in the title or the abstract | 1 |
|  |  | (*b*) Provide in the abstract an informative and balanced summary of what was done and what was found | 3 |
| **Introduction** |  |  |  |
| Background/rationale | 2 | Explain the scientific background and rationale for the investigation being reported | 5-6 |
| Objectives | 3 | State specific objectives, including any prespecified hypotheses | 6 |
| **Methods** |  |  |  |
| Study design | 4 | Present key elements of study design early in the paper | 6 |
| Setting | 5 | Describe the setting, locations, and relevant dates, including periods of recruitment, exposure, follow-up, and data collection | 6 |
| Participants | 6 | (*a*) *Cohort study*—Give the eligibility criteria, and the sources and methods of selection of participants. Describe methods of follow-up  *Case-control study*—Give the eligibility criteria, and the sources and methods of case ascertainment and control selection. Give the rationale for the choice of cases and controls  *Cross-sectional study*—Give the eligibility criteria, and the sources and methods of selection of participants | 6 |
|  |  | (*b*) *Cohort study*—For matched studies, give matching criteria and number of exposed and unexposed  *Case-control study*—For matched studies, give matching criteria and the number of controls per case | – |
| Variables | 7 | Clearly define all outcomes, exposures, predictors, potential confounders, and effect modifiers. Give diagnostic criteria, if applicable | 6-8 |
| Data sources/ measurement | 8 | For each variable of interest, give sources of data and details of methods of assessment (measurement). Describe comparability of assessment methods if there is more than one group | 6-8, Suppl. Methods |
| Bias | 9 | Describe any efforts to address potential sources of bias | 8-11 |
| Study size | 10 | Explain how the study size was arrived at | 11, Suppl. Methods |
| Quantitative variables | 11 | Explain how quantitative variables were handled in the analyses. If applicable, describe which groupings were chosen and why | 8-11, Suppl. Methods |
| Statistical methods | 12 | (*a*) Describe all statistical methods, including those used to control for confounding | 8-11 |
|  |  | (*b*) Describe any methods used to examine subgroups and interactions | 9-10 |
|  |  | (*c*) Explain how missing data were addressed | 8, Suppl. Methods |
|  |  | (*d*) *Cohort study*—If applicable, explain how loss to follow-up was addressed  *Case-control study*—If applicable, explain how matching of cases and controls was addressed  *Cross-sectional study*—If applicable, describe analytical methods taking account of sampling strategy | 8-9 |
|  |  | (*e*) Describe any sensitivity analyses | 9-11 |
| **Results** |  |  |  |
| Participants | 13 | (a) Report numbers of individuals at each stage of study—eg numbers potentially eligible, examined for eligibility, confirmed eligible, included in the study, completing follow-up, and analysed | 11, Fig. 1, Suppl. Methods |
|  |  | (b) Give reasons for non-participation at each stage | Fig. 1, Suppl. Methods |
|  |  | (c) Consider use of a flow diagram | Fig. 1 |
| Descriptive data | 14 | (a) Give characteristics of study participants (eg demographic, clinical, social) and information on exposures and potential confounders | 11-12, Table 1 |
|  |  | (b) Indicate number of participants with missing data for each variable of interest | Table 1 |
|  |  | (c) *Cohort study*—Summarise follow-up time (eg, average and total amount) | 12 |
| Outcome data | 15 | *Cohort study*—Report numbers of outcome events or summary measures over time | 12 |
|  |  | *Case-control study—*Report numbers in each exposure category, or summary measures of exposure | – |
|  |  | *Cross-sectional study—*Report numbers of outcome events or summary measures | – |
| Main results | 16 | (*a*) Give unadjusted estimates and, if applicable, confounder-adjusted estimates and their precision (eg, 95% confidence interval). Make clear which confounders were adjusted for and why they were included | 12, Fig.2, Table S4 |
|  |  | (*b*) Report category boundaries when continuous variables were categorized | 12, Fig.2, Table S4 |
|  |  | (*c*) If relevant, consider translating estimates of relative risk into absolute risk for a meaningful time period | – |
| Other analyses | 17 | Report other analyses done—eg analyses of subgroups and interactions, and sensitivity analyses | 12, Table S5 |
| **Discussion** |  |  |  |
| Key results | 18 | Summarise key results with reference to study objectives | 13-14 |
| Limitations | 19 | Discuss limitations of the study, taking into account sources of potential bias or imprecision. Discuss both direction and magnitude of any potential bias | 17-20 |
| Interpretation | 20 | Give a cautious overall interpretation of results considering objectives, limitations, multiplicity of analyses, results from similar studies, and other relevant evidence | 20 |
| Generalisability | 21 | Discuss the generalisability (external validity) of the study results | 17-20 |
| **Other information** |  |  |  |
| Funding | 22 | Give the source of funding and the role of the funders for the present study and, if applicable, for the original study on which the present article is based | 21-23 |

Table S2. STROBE-MR checklist.

| **Item No.** | **Section** | **Checklist item** | **Page No.** |
| --- | --- | --- | --- |
| 1 | Title and abstract | Indicate Mendelian randomization (MR) as the study’s design in the title and/or the abstract if that is a main purpose of the study | 1, 3 |
|  | **Introduction** |  |  |
| 2 | Background | Explain the scientific background and rationale for the reported study. What is the exposure? Is a potential causal relationship between exposure and outcome plausible? Justify why MR is a helpful method to address the study question | 5-6 |
| 3 | Objectives | State specific objectives clearly, including pre-specified causal hypotheses (if any). State that MR is a method that, under specific assumptions, intends to estimate causal effects | 5-6 |
|  | **Methods** |  |  |
| 4 | Study design and data sources | Present key elements of the study design early in the article. Consider including a table listing sources of data for all phases of the study. For each data source contributing to the analysis, describe the following: | 6 |
|  | a) | Setting: Describe the study design and the underlying population, if possible. Describe the setting, locations, and relevant dates, including periods of recruitment, exposure, follow-up, and data collection, when available. | 6 |
|  | b) | Participants: Give the eligibility criteria, and the sources and methods of selection of participants. Report the sample size, and whether any power or sample size calculations were carried out prior to the main analysis | 6 |
|  | c) | Describe measurement, quality control and selection of genetic variants | 7 |
|  | d) | For each exposure, outcome, and other relevant variables, describe methods of assessment and diagnostic criteria for diseases | 6-8, Suppl. Methods |
|  | e) | Provide details of ethics committee approval and participant informed consent, if relevant | 21 |
| 5 | Assumptions | Explicitly state the three core IV assumptions for the main analysis (relevance, independence and exclusion restriction) as well assumptions for any additional or sensitivity analysis | 19 |
| 6 | Statistical methods: main analysis | Describe statistical methods and statistics used |  |
|  | a) | Describe how quantitative variables were handled in the analyses (i.e., scale, units, model) | 8-11 |
|  | b) | Describe how genetic variants were handled in the analyses and, if applicable, how their weights were selected | 7, 9-11 |
|  | c) | Describe the MR estimator (e.g. two-stage least squares, Wald ratio) and related statistics. Detail the included covariates and, in case of two-sample MR, whether the same covariate set was used for adjustment in the two samples | 9-11 |
|  | d) | Explain how missing data were addressed | 6-8 |
|  | e) | If applicable, indicate how multiple testing was addressed | – |
| 7 | Assessment of assumptions | Describe any methods or prior knowledge used to assess the assumptions or justify their validity | 9-11 |
| 8 | Sensitivity analyses and additional analyses | Describe any sensitivity analyses or additional analyses performed (e.g. comparison of effect estimates from different approaches, independent replication, bias analytic techniques, validation of instruments, simulations) | 9-11 |
| 9 | Software and pre-registration |  |  |
|  | a) | Name statistical software and package(s), including version and settings used | 8-11 |
|  | b) | State whether the study protocol and details were pre-registered (as well as when and where) | – |
|  | **Results** |  |  |
| 10 | Descriptive data |  |  |
|  | a) | Report the numbers of individuals at each stage of included studies and reasons for exclusion. Consider use of a flow diagram | 11, Fig. 1, Suppl. Methods |
|  | b) | Report summary statistics for phenotypic exposure(s), outcome(s), and other relevant variables (e.g. means, SDs, proportions) | 11-12, Table 1 |
|  | c) | If the data sources include meta-analyses of previous studies, provide the assessments of heterogeneity across these studies | doi: 10.1038/ng.3841 |
|  | d) | For two-sample MR:  i.  Provide justification of the similarity of the genetic variant-exposure associations between the exposure and outcome samples  ii.  Provide information on the number of individuals who overlap between the exposure and outcome studies | 18-19 |
| 11 | Main results |  |  |
|  | a) | Report the associations between genetic variant and exposure, and between genetic variant and outcome, preferably on an interpretable scale | doi: 10.1038/ng.3841, Table S3 |
|  | b) | Report MR estimates of the relationship between exposure and outcome, and the measures of uncertainty from the MR analysis, on an interpretable scale, such as odds ratio or relative risk per SD difference | 12-13, Fig.3, Figure S2-S5 |
|  | c) | If relevant, consider translating estimates of relative risk into absolute risk for a meaningful time period | – |
|  | d) | Consider plots to visualize results (e.g. forest plot, scatterplot of associations between genetic variants and outcome versus between genetic variants and exposure) | Fig. 3, Figure S2-S5 |
| 12 | Assessment of assumptions |  |  |
|  | a) | Report the assessment of the validity of the assumptions | 12-13, 19-20 |
|  | b) | Report any additional statistics (e.g., assessments of heterogeneity across genetic variants, such as *I^2^*, Q statistic or E-value) | 12-13 |
| 13 | Sensitivity analyses and additional analyses |  |  |
|  | a) | Report any sensitivity analyses to assess the robustness of the main results to violations of the assumptions | 13 |
|  | b) | Report results from other sensitivity analyses or additional analyses | 13 |
|  | c) | Report any assessment of direction of causal relationship (e.g., bidirectional MR) | 13 |
|  | d) | When relevant, report and compare with estimates from non-MR analyses | 12 |
|  | e) | Consider additional plots to visualize results (e.g., leave-one-out analyses) | – |
|  | **Discussion** |  |  |
| 14 | Key results | Summarize key results with reference to study objectives | 13-14 |
| 15 | Limitations | Discuss limitations of the study, taking into account the validity of the IV assumptions, other sources of potential bias, and imprecision. Discuss both direction and magnitude of any potential bias and any efforts to address them | 17-20 |
| 16 | Interpretation |  |  |
|  | a) | Meaning: Give a cautious overall interpretation of results in the context of their limitations and in comparison with other studies | 20 |
|  | b) | Mechanism: Discuss underlying biological mechanisms that could drive a potential causal relationship between the investigated exposure and the outcome, and whether the gene-environment equivalence assumption is reasonable. Use causal language carefully, clarifying that IV estimates may provide causal effects only under certain assumptions | 15-17 |
|  | c) | Clinical relevance: Discuss whether the results have clinical or public policy relevance, and to what extent they inform effect sizes of possible interventions | 16-17 |
| 17 | Generalizability | Discuss the generalizability of the study results (a) to other populations, (b) across other exposure periods/timings, and (c) across other levels of exposure | 17-20 |
|  | **Other Information** |  |  |
| 18 | Funding | Describe sources of funding and the role of funders in the present study and, if applicable, sources of funding for the databases and original study or studies on which the present study is based | 21-23 |
| 19 | Data and data sharing | Provide the data used to perform all analyses or report where and how the data can be accessed, and reference these sources in the article. Provide the statistical code needed to reproduce the results in the article, or report whether the code is publicly accessible and if so, where | 21, doi: 10.1038/ng.3841, UK Biobank website |
| 20 | Conflicts of Interest | All authors should declare all potential conflicts of interest | 21 |

Table S3. Single nucleotide polymorphisms included in the genetic instruments and their association with cardiovascular events.

|  |  |  |  |  |  |  |  | **Myocardial infarction** | | | | **Ischaemic stroke** | | | | **Haemorrhagic stroke** | | | |
| --- | --- | --- | --- | --- | --- | --- | --- | --- | --- | --- | --- | --- | --- | --- | --- | --- | --- | --- | --- |
|  |  |  |  |  |  |  |  | **EPIC-CVD** | | **UK Biobank** | | **EPIC-CVD** | | **UK Biobank** | | **EPIC-CVD** | | **UK Biobank** | |
| **rsid** | **Chr** | **Position** | **EA** | **OA** | **Proxy (HCE)** | **Proxy (OEE)** | **Proxy (Quad)** | **beta** | **SE** | **beta** | **SE** | **beta** | **SE** | **beta** | **SE** | **beta** | **SE** | **beta** | **SE** |
| rs6678140 | 1 | 8436802 | T | C | - | - | - | -3.94e-02 | 5.85e-02 | 7.73e-03 | 2.27e-02 | -6.14e-02 | 5.85e-02 | 6.87e-03 | 2.85e-02 | -4.93e-04 | 7.50e-02 | 5.70e-02 | 4.61e-02 |
| rs141847393 | 1 | 27212209 | T | C | - | - | - | -8.82e-02 | 9.73e-02 | -4.13e-02 | 3.88e-02 | 2.93e-03 | 1.05e-01 | -2.17e-02 | 4.92e-02 | -2.11e-01 | 1.26e-01 | -9.40e-03 | 7.91e-02 |
| rs360495 | 1 | 33228197 | A | T | - | - | - | 1.04e-02 | 9.42e-02 | -4.25e-02 | 3.94e-02 | 4.55e-02 | 9.08e-02 | -6.11e-02 | 5.01e-02 | -3.97e-02 | 1.25e-01 | 6.60e-03 | 7.79e-02 |
| rs11209331 | 1 | 41456689 | T | C | - | - | - | 8.69e-03 | 5.72e-02 | 1.24e-02 | 2.17e-02 | -3.98e-02 | 5.59e-02 | 2.27e-02 | 2.74e-02 | 1.06e-01 | 7.46e-02 | 1.08e-02 | 4.38e-02 |
| rs11210871 | 1 | 44029353 | C | G | - | - | - | -8.45e-02 | 6.09e-02 | 4.38e-03 | 2.33e-02 | -2.68e-02 | 5.89e-02 | -1.99e-02 | 2.95e-02 | -7.49e-02 | 7.55e-02 | -3.91e-02 | 4.74e-02 |
| rs643428 | 1 | 54728858 | T | C | - | - | - | -8.44e-02 | 5.72e-02 | -1.92e-02 | 2.21e-02 | -4.55e-02 | 5.64e-02 | 1.55e-02 | 2.79e-02 | 6.86e-02 | 7.72e-02 | 1.16e-02 | 4.46e-02 |
| rs7516763 | 1 | 65972550 | A | C | - | - | - | 9.34e-02 | 5.62e-02 | 2.06e-02 | 2.17e-02 | 1.42e-01 | 5.65e-02 | -3.27e-02 | 2.74e-02 | 1.88e-01 | 7.31e-02 | -6.20e-02 | 4.38e-02 |
| rs11209943 | 1 | 72750500 | A | G | - | - | - | 2.48e-02 | 6.12e-02 | -1.37e-02 | 2.20e-02 | -4.50e-03 | 5.93e-02 | -2.15e-02 | 2.77e-02 | -3.79e-02 | 7.87e-02 | 2.09e-02 | 4.42e-02 |
| rs1040070 | 1 | 74977870 | C | G | - | - | - | 6.54e-02 | 5.58e-02 | -9.37e-03 | 2.18e-02 | 1.04e-01 | 5.54e-02 | 3.54e-02 | 2.76e-02 | 4.55e-02 | 7.23e-02 | -9.10e-02 | 4.38e-02 |
| rs10782777 | 1 | 82562929 | A | G | - | - | - | -7.32e-02 | 6.02e-02 | 1.67e-02 | 2.24e-02 | -3.12e-02 | 5.94e-02 | 3.38e-02 | 2.82e-02 | -8.64e-02 | 7.85e-02 | 4.61e-03 | 4.50e-02 |
| rs11165924 | 1 | 98375448 | A | G | - | - | - | 6.51e-02 | 6.66e-02 | -3.16e-04 | 2.31e-02 | 3.69e-02 | 6.20e-02 | 1.90e-02 | 2.92e-02 | 4.10e-02 | 8.15e-02 | -3.74e-03 | 4.65e-02 |
| rs4561063 | 1 | 102520898 | T | G | - | - | - | -3.30e-02 | 5.53e-02 | 1.82e-02 | 2.15e-02 | -1.56e-04 | 5.51e-02 | -2.29e-02 | 2.71e-02 | -7.58e-02 | 6.93e-02 | -4.42e-02 | 4.34e-02 |
| rs61817552 | 1 | 150423577 | A | G | - | - | - | 9.48e-02 | 7.13e-02 | 1.41e-02 | 2.59e-02 | 4.40e-02 | 6.81e-02 | -2.94e-03 | 3.27e-02 | -5.61e-02 | 8.59e-02 | -7.11e-02 | 5.33e-02 |
| rs6661100 | 1 | 150758727 | T | C | - | - | - | 1.02e-01 | 9.78e-02 | -2.77e-02 | 3.99e-02 | 5.44e-02 | 9.88e-02 | -8.18e-02 | 5.14e-02 | -6.11e-02 | 1.37e-01 | -1.47e-01 | 8.44e-02 |
| rs4845364 | 1 | 154141908 | A | G | - | - | - | 1.22e-02 | 5.66e-02 | 1.57e-02 | 2.15e-02 | 4.34e-03 | 5.67e-02 | 1.11e-02 | 2.71e-02 | -1.03e-02 | 6.96e-02 | 2.58e-04 | 4.33e-02 |
| rs9427116 | 1 | 154631123 | T | C | - | - | - | 1.01e-01 | 5.67e-02 | -8.26e-03 | 2.14e-02 | 3.36e-02 | 5.69e-02 | 2.51e-02 | 2.69e-02 | 3.13e-02 | 7.07e-02 | 1.16e-02 | 4.31e-02 |
| rs2343507 | 1 | 162895515 | A | C | - | - | - | 2.94e-02 | 5.78e-02 | 2.79e-02 | 2.20e-02 | -9.82e-03 | 5.69e-02 | -1.76e-02 | 2.76e-02 | 1.11e-01 | 7.42e-02 | 2.51e-02 | 4.44e-02 |
| rs2661339 | 1 | 163018934 | T | G | - | - | - | 7.90e-02 | 1.23e-01 | 1.38e-02 | 4.91e-02 | 9.41e-02 | 1.20e-01 | 1.88e-02 | 6.15e-02 | 4.32e-02 | 1.54e-01 | 5.92e-02 | 9.70e-02 |
| rs157877 | 1 | 165398744 | A | G | - | - | - | 7.24e-02 | 8.32e-02 | 1.54e-02 | 3.20e-02 | 1.52e-01 | 7.78e-02 | -5.66e-02 | 4.15e-02 | -2.05e-02 | 1.08e-01 | 3.81e-02 | 6.43e-02 |
| rs506589 | 1 | 177894287 | T | C | - | - | - | -9.83e-02 | 7.02e-02 | 5.68e-03 | 2.65e-02 | -7.77e-02 | 6.85e-02 | -1.47e-02 | 3.31e-02 | -6.31e-02 | 8.94e-02 | -7.50e-03 | 5.32e-02 |
| rs61828391 | 1 | 179732142 | A | G | - | - | - | -1.29e-01 | 8.31e-02 | -2.82e-02 | 3.30e-02 | -1.08e-01 | 8.35e-02 | -3.32e-02 | 4.16e-02 | -2.00e-01 | 1.05e-01 | 4.72e-02 | 6.47e-02 |
| rs11240695 | 1 | 204158132 | A | C | - | - | - | -2.72e-02 | 6.72e-02 | 2.48e-04 | 2.48e-02 | -9.72e-02 | 6.99e-02 | -4.44e-02 | 3.15e-02 | -9.61e-02 | 8.57e-02 | -4.23e-02 | 5.04e-02 |
| rs4951261 | 1 | 205717823 | A | C | - | - | - | 2.30e-03 | 5.73e-02 | 2.09e-02 | 2.20e-02 | 5.09e-02 | 5.82e-02 | 2.32e-03 | 2.76e-02 | 1.22e-01 | 7.42e-02 | 3.15e-02 | 4.43e-02 |
| rs12040029 | 1 | 213451958 | T | C | - | - | - | -1.78e-02 | 9.01e-02 | -4.14e-02 | 3.50e-02 | 4.04e-02 | 8.68e-02 | 1.84e-05 | 4.34e-02 | 1.16e-01 | 1.17e-01 | -2.30e-02 | 7.00e-02 |
| rs7576624 | 2 | 625029 | T | C | - | - | - | 4.68e-02 | 7.29e-02 | 5.47e-02 | 2.89e-02 | 4.45e-02 | 7.37e-02 | -8.18e-03 | 3.57e-02 | -4.11e-03 | 9.67e-02 | 6.51e-02 | 5.85e-02 |
| rs7587651 | 2 | 10368606 | T | C | - | - | - | 1.30e-02 | 6.02e-02 | -1.83e-02 | 2.24e-02 | -1.87e-02 | 5.70e-02 | -2.69e-02 | 2.83e-02 | 1.24e-02 | 7.32e-02 | -8.47e-02 | 4.56e-02 |
| rs150821390 | 2 | 24106445 | T | C | - | - | - | -1.63e-01 | 1.93e-01 | -2.13e-01 | 7.41e-02 | 1.30e-01 | 1.67e-01 | 1.31e-01 | 7.98e-02 | -1.46e-01 | 2.44e-01 | -7.97e-02 | 1.41e-01 |
| rs72787511 | 2 | 32816089 | C | G | - | - | - | -3.91e-01 | 1.74e-01 | 6.39e-04 | 5.78e-02 | -3.00e-01 | 1.73e-01 | 4.79e-03 | 7.25e-02 | -6.31e-01 | 2.56e-01 | -2.92e-01 | 1.33e-01 |
| rs10175423 | 2 | 42970161 | T | C | - | - | - | -3.85e-02 | 6.03e-02 | -1.47e-02 | 2.37e-02 | 4.97e-02 | 5.90e-02 | 2.39e-02 | 2.96e-02 | 2.10e-02 | 7.78e-02 | 6.45e-02 | 4.70e-02 |
| rs17390720 | 2 | 44952254 | C | G | - | - | - | 5.67e-02 | 6.64e-02 | -1.26e-02 | 2.48e-02 | 1.34e-01 | 6.59e-02 | -1.50e-03 | 3.12e-02 | 1.55e-02 | 8.31e-02 | -2.20e-03 | 4.99e-02 |
| rs111567162 | 2 | 56588406 | A | T | rs6545574 | - | rs6545574 | 1.04e-02 | 7.18e-02 | 3.34e-02 | 2.86e-02 | -3.23e-02 | 7.32e-02 | -1.84e-02 | 3.65e-02 | -6.11e-02 | 9.30e-02 | 1.24e-01 | 5.60e-02 |
| rs1025128 | 2 | 60175475 | C | G | - | - | - | -3.37e-02 | 5.68e-02 | -6.21e-03 | 2.17e-02 | -2.45e-02 | 5.61e-02 | 5.09e-02 | 2.74e-02 | 4.76e-02 | 7.50e-02 | 1.50e-02 | 4.37e-02 |
| rs10205969 | 2 | 61367664 | T | C | - | - | - | -2.97e-02 | 8.39e-02 | 1.55e-02 | 3.01e-02 | 4.54e-02 | 8.11e-02 | -1.86e-02 | 3.84e-02 | 2.22e-02 | 1.07e-01 | 4.20e-02 | 6.01e-02 |
| rs12467441 | 2 | 61685826 | T | C | - | - | - | -6.46e-02 | 8.72e-02 | -2.12e-02 | 3.26e-02 | -8.95e-02 | 8.33e-02 | -6.81e-03 | 4.12e-02 | 1.93e-02 | 1.15e-01 | -3.61e-02 | 6.52e-02 |
| rs2723065 | 2 | 65279414 | A | G | - | - | - | 2.71e-02 | 6.00e-02 | 4.10e-02 | 2.22e-02 | 3.06e-02 | 5.95e-02 | 5.29e-02 | 2.79e-02 | -9.03e-02 | 7.39e-02 | 5.08e-03 | 4.45e-02 |
| rs2312205 | 2 | 69704941 | A | G | - | - | - | 3.61e-02 | 7.40e-02 | -2.20e-02 | 2.74e-02 | -9.33e-02 | 7.19e-02 | -1.71e-02 | 3.45e-02 | -8.22e-02 | 9.96e-02 | 8.36e-02 | 5.71e-02 |
| rs34437050 | 2 | 73535526 | A | G | - | - | - | -5.42e-01 | 2.71e-01 | -1.00e-01 | 9.43e-02 | -5.04e-01 | 2.70e-01 | 1.16e-01 | 1.07e-01 | -5.20e-01 | 3.93e-01 | -3.00e-01 | 2.09e-01 |
| rs2558101 | 2 | 137613322 | A | G | - | - | - | 3.54e-02 | 6.28e-02 | -6.03e-02 | 2.30e-02 | -2.31e-02 | 6.08e-02 | -2.75e-02 | 2.91e-02 | -3.87e-02 | 7.94e-02 | -4.15e-02 | 4.64e-02 |
| rs35935052 | 2 | 142302503 | T | G | - | - | - | -1.56e-02 | 8.30e-02 | 1.81e-02 | 2.95e-02 | 1.33e-03 | 7.94e-02 | -8.06e-02 | 3.86e-02 | 9.75e-02 | 1.03e-01 | 5.48e-02 | 5.89e-02 |
| rs6434162 | 2 | 153556801 | A | G | - | - | - | -4.52e-02 | 7.53e-02 | 2.39e-02 | 2.81e-02 | 3.69e-02 | 7.34e-02 | -5.60e-02 | 3.44e-02 | 6.88e-02 | 9.66e-02 | -2.65e-02 | 5.58e-02 |
| rs142058842 | 2 | 156621725 | C | G | - | - | - | 3.04e-02 | 7.34e-02 | 2.63e-02 | 2.94e-02 | 5.82e-02 | 7.28e-02 | -3.14e-02 | 3.63e-02 | 5.80e-02 | 9.63e-02 | -2.23e-02 | 5.83e-02 |
| rs145438026 | 2 | 157228255 | T | C | - | - | - | 3.38e-02 | 1.17e-01 | 3.98e-02 | 4.36e-02 | 6.50e-02 | 1.09e-01 | -3.09e-02 | 5.65e-02 | -5.71e-02 | 1.48e-01 | 1.42e-03 | 8.91e-02 |
| rs2271758 | 2 | 172701157 | T | G | - | - | - | -7.02e-02 | 5.76e-02 | -1.63e-02 | 2.19e-02 | -3.67e-02 | 5.67e-02 | 1.55e-02 | 2.75e-02 | -5.47e-02 | 7.72e-02 | -1.24e-02 | 4.41e-02 |
| rs842567 | 2 | 184291116 | A | C | - | - | - | 7.78e-02 | 7.31e-02 | -4.28e-02 | 2.64e-02 | 1.29e-01 | 7.10e-02 | -2.77e-02 | 3.34e-02 | 1.81e-01 | 9.68e-02 | -2.45e-02 | 5.35e-02 |
| rs13023912 | 2 | 199756278 | A | G | - | - | - | -3.94e-02 | 5.87e-02 | -2.21e-02 | 2.25e-02 | -1.08e-02 | 5.73e-02 | -2.61e-02 | 2.83e-02 | -1.22e-02 | 7.70e-02 | -7.76e-02 | 4.50e-02 |
| rs16841867 | 2 | 203168235 | C | G | - | - | - | 1.13e-01 | 8.89e-02 | -2.44e-02 | 3.29e-02 | -1.17e-02 | 8.98e-02 | -9.33e-03 | 4.19e-02 | 1.38e-01 | 1.17e-01 | 1.12e-01 | 6.98e-02 |
| rs184033703 | 2 | 206956138 | A | G | - | - | - | -2.73e-03 | 1.25e-01 | -8.31e-02 | 4.86e-02 | 1.56e-02 | 1.25e-01 | -6.75e-02 | 6.07e-02 | 2.99e-03 | 1.67e-01 | 1.12e-01 | 8.97e-02 |
| rs6735626 | 2 | 213403972 | A | G | - | - | - | 2.24e-02 | 5.56e-02 | 3.39e-02 | 2.16e-02 | 4.66e-02 | 5.43e-02 | -2.86e-03 | 2.73e-02 | 2.02e-02 | 7.04e-02 | 9.07e-02 | 4.35e-02 |
| rs73820560 | 3 | 1906245 | A | C | - | - | - | 9.30e-03 | 8.71e-02 | -9.46e-03 | 3.08e-02 | -1.35e-01 | 8.14e-02 | 1.04e-01 | 4.04e-02 | 1.39e-01 | 1.08e-01 | -1.56e-01 | 5.91e-02 |
| rs9867904 | 3 | 18442437 | C | G | - | - | - | 2.18e-02 | 5.72e-02 | -1.41e-02 | 2.19e-02 | 8.37e-02 | 5.68e-02 | -1.86e-02 | 2.77e-02 | -3.28e-02 | 7.33e-02 | 5.21e-02 | 4.39e-02 |
| rs73035994 | 3 | 24206463 | T | C | - | - | - | 6.43e-02 | 1.85e-01 | 9.20e-02 | 6.72e-02 | 1.93e-02 | 1.92e-01 | -2.83e-02 | 8.01e-02 | -1.31e-01 | 2.31e-01 | -7.06e-02 | 1.26e-01 |
| rs1984870 | 3 | 24715135 | T | G | - | - | - | 4.32e-02 | 5.48e-02 | 7.50e-03 | 2.17e-02 | -2.20e-02 | 5.48e-02 | -9.34e-04 | 2.73e-02 | -1.49e-03 | 7.30e-02 | -2.40e-03 | 4.38e-02 |
| rs77955256 | 3 | 44883523 | A | T | - | - | - | -1.30e-02 | 8.98e-02 | 5.78e-03 | 3.45e-02 | 8.81e-03 | 9.14e-02 | 2.51e-02 | 4.30e-02 | -4.33e-02 | 1.21e-01 | 2.74e-03 | 6.96e-02 |
| rs6803264 | 3 | 49254427 | T | C | - | - | - | -1.92e-02 | 6.84e-02 | 7.95e-02 | 2.58e-02 | -2.48e-02 | 6.56e-02 | 2.37e-02 | 3.29e-02 | 1.07e-01 | 8.31e-02 | 1.62e-02 | 5.28e-02 |
| rs115435316 | 3 | 49568181 | A | G | - | - | - | -2.24e-01 | 1.42e-01 | -1.79e-01 | 6.38e-02 | -1.95e-01 | 1.49e-01 | -1.11e-01 | 7.77e-02 | -6.57e-02 | 1.84e-01 | -8.79e-02 | 1.23e-01 |
| rs6445624 | 3 | 51358019 | A | G | - | - | - | -1.57e-01 | 8.46e-02 | -4.04e-02 | 3.16e-02 | -1.35e-01 | 8.19e-02 | 4.88e-02 | 3.85e-02 | -1.25e-02 | 1.03e-01 | 2.03e-02 | 6.22e-02 |
| rs10933 | 3 | 52719816 | T | C | - | - | - | -5.76e-02 | 5.86e-02 | -1.70e-02 | 2.16e-02 | 4.74e-02 | 5.68e-02 | 1.51e-03 | 2.71e-02 | 3.04e-02 | 7.25e-02 | 3.18e-02 | 4.34e-02 |
| rs7431217 | 3 | 68595634 | T | C | - | - | - | -1.07e-01 | 5.94e-02 | -1.40e-02 | 2.19e-02 | -1.04e-01 | 6.00e-02 | -2.81e-02 | 2.76e-02 | -3.92e-02 | 7.49e-02 | 1.45e-02 | 4.41e-02 |
| rs7426534 | 3 | 84462073 | A | G | - | - | - | 7.68e-02 | 6.39e-02 | 6.28e-03 | 2.36e-02 | 7.27e-02 | 6.19e-02 | -3.80e-02 | 2.94e-02 | 1.39e-01 | 8.09e-02 | 3.95e-03 | 4.75e-02 |
| rs9758500 | 3 | 86910329 | A | G | - | - | - | 1.94e-02 | 5.71e-02 | 9.60e-03 | 2.21e-02 | -3.43e-02 | 5.77e-02 | -6.10e-02 | 2.81e-02 | 7.65e-02 | 7.37e-02 | 6.36e-02 | 4.42e-02 |
| rs4859001 | 3 | 88221517 | T | C | - | - | - | 2.22e-02 | 8.20e-02 | 1.02e-02 | 2.92e-02 | 1.83e-02 | 7.98e-02 | 5.30e-03 | 3.66e-02 | 5.68e-02 | 1.03e-01 | -2.57e-03 | 5.89e-02 |
| rs709488 | 3 | 107700952 | A | C | - | - | - | 2.05e-02 | 5.61e-02 | -2.59e-03 | 2.14e-02 | 9.55e-03 | 5.52e-02 | 2.40e-02 | 2.70e-02 | -1.44e-03 | 7.13e-02 | 1.99e-02 | 4.32e-02 |
| rs9834893 | 3 | 114574749 | C | G | - | - | - | 2.28e-01 | 1.09e-01 | 4.30e-02 | 4.29e-02 | 9.73e-02 | 1.06e-01 | -7.20e-02 | 5.15e-02 | 1.22e-01 | 1.35e-01 | 7.73e-02 | 8.79e-02 |
| rs10934420 | 3 | 117552111 | T | C | - | - | - | -2.89e-03 | 5.59e-02 | 2.08e-03 | 2.14e-02 | 2.53e-03 | 5.44e-02 | -1.58e-02 | 2.70e-02 | 3.89e-02 | 7.13e-02 | 5.12e-02 | 4.32e-02 |
| rs2461794 | 3 | 127870060 | A | G | - | - | - | -7.94e-03 | 6.40e-02 | -4.43e-02 | 2.43e-02 | 1.21e-02 | 6.18e-02 | 7.17e-03 | 3.02e-02 | 4.17e-02 | 8.05e-02 | -9.93e-02 | 4.97e-02 |
| rs6439371 | 3 | 132610752 | A | G | - | - | - | -1.19e-01 | 5.84e-02 | 3.94e-02 | 2.25e-02 | -7.84e-02 | 5.76e-02 | 7.04e-03 | 2.82e-02 | -1.48e-02 | 7.12e-02 | -4.85e-02 | 4.48e-02 |
| rs6439713 | 3 | 137128815 | A | C | - | - | - | -4.27e-02 | 6.13e-02 | 1.82e-02 | 2.29e-02 | -1.31e-02 | 6.08e-02 | -1.99e-02 | 2.90e-02 | -2.82e-02 | 7.94e-02 | -2.65e-02 | 4.65e-02 |
| rs11711674 | 3 | 156532953 | T | C | - | - | - | 1.62e-02 | 5.73e-02 | 5.53e-03 | 2.17e-02 | -3.96e-03 | 5.55e-02 | 6.74e-03 | 2.74e-02 | -8.58e-02 | 6.96e-02 | -3.73e-02 | 4.37e-02 |
| rs13322435 | 3 | 156795468 | A | G | - | - | - | 6.67e-03 | 5.76e-02 | 1.73e-02 | 2.20e-02 | 1.18e-01 | 5.99e-02 | 9.60e-03 | 2.77e-02 | 5.69e-02 | 7.55e-02 | -2.65e-02 | 4.42e-02 |
| rs582780 | 3 | 172121443 | A | G | - | - | - | 5.57e-02 | 5.75e-02 | 5.79e-03 | 2.19e-02 | 7.42e-02 | 5.73e-02 | -4.65e-03 | 2.75e-02 | 1.72e-01 | 7.42e-02 | -1.13e-01 | 4.38e-02 |
| rs2300922 | 3 | 185651469 | T | C | - | - | - | 3.86e-03 | 5.66e-02 | -6.25e-03 | 2.18e-02 | 2.02e-02 | 5.56e-02 | 2.54e-02 | 2.74e-02 | 1.24e-02 | 7.54e-02 | 2.21e-02 | 4.39e-02 |
| rs2108753 | 4 | 3266860 | T | C | - | - | - | 4.87e-03 | 5.71e-02 | 7.35e-03 | 2.17e-02 | -1.89e-02 | 5.62e-02 | 1.65e-02 | 2.74e-02 | -4.92e-02 | 7.17e-02 | -9.34e-03 | 4.37e-02 |
| rs4340786 | 4 | 28746246 | A | T | - | - | - | 1.35e-01 | 6.40e-02 | -3.20e-02 | 2.43e-02 | 1.11e-01 | 6.74e-02 | 2.34e-03 | 3.09e-02 | -1.63e-02 | 8.41e-02 | 3.14e-02 | 4.99e-02 |
| rs4588499 | 4 | 45910674 | A | G | - | - | - | 9.22e-02 | 5.88e-02 | 2.31e-03 | 2.15e-02 | 1.66e-02 | 5.52e-02 | 5.82e-02 | 2.71e-02 | 1.60e-02 | 7.33e-02 | -1.27e-02 | 4.34e-02 |
| rs3113862 | 4 | 95143122 | A | G | - | - | - | -4.19e-02 | 5.62e-02 | 2.41e-02 | 2.19e-02 | 3.56e-02 | 5.64e-02 | 2.05e-02 | 2.76e-02 | -5.51e-02 | 7.36e-02 | -3.92e-02 | 4.39e-02 |
| rs55784701 | 4 | 104247262 | T | C | - | - | - | 7.80e-02 | 6.14e-02 | 6.13e-03 | 2.61e-02 | 6.36e-02 | 6.19e-02 | 5.64e-02 | 3.23e-02 | 1.33e-01 | 8.05e-02 | -3.83e-02 | 5.31e-02 |
| rs3733632 | 4 | 104640935 | A | G | - | - | - | 4.21e-02 | 7.87e-02 | 5.18e-02 | 2.99e-02 | 1.32e-02 | 7.88e-02 | 3.70e-03 | 3.72e-02 | 5.72e-02 | 1.06e-01 | -3.47e-02 | 5.88e-02 |
| rs17035311 | 4 | 106066293 | A | C | - | - | - | 1.40e-01 | 8.38e-02 | 3.91e-02 | 3.09e-02 | 3.07e-02 | 7.89e-02 | -1.66e-02 | 3.82e-02 | 2.46e-02 | 1.07e-01 | 3.38e-02 | 6.23e-02 |
| rs62316795 | 4 | 132621869 | A | C | - | - | - | 5.59e-03 | 6.86e-02 | 1.47e-02 | 2.70e-02 | 3.35e-02 | 6.87e-02 | -2.18e-02 | 3.43e-02 | 1.89e-02 | 9.12e-02 | -8.71e-03 | 5.46e-02 |
| rs13120031 | 4 | 177465182 | T | C | - | - | - | -6.91e-02 | 6.20e-02 | 1.11e-02 | 2.33e-02 | -4.01e-02 | 5.88e-02 | 7.94e-02 | 2.90e-02 | -1.65e-02 | 7.66e-02 | -1.15e-02 | 4.72e-02 |
| rs10521021 | 5 | 35030311 | T | G | - | - | - | -4.61e-02 | 5.89e-02 | -2.00e-02 | 2.29e-02 | -4.76e-02 | 5.77e-02 | -2.18e-02 | 2.88e-02 | -1.23e-01 | 7.51e-02 | -8.63e-02 | 4.56e-02 |
| rs62361685 | 5 | 41994067 | T | C | - | - | - | 6.33e-02 | 1.27e-01 | 1.32e-02 | 4.56e-02 | 1.41e-01 | 1.41e-01 | 3.64e-02 | 5.79e-02 | 3.41e-01 | 1.86e-01 | 1.62e-01 | 9.82e-02 |
| rs7712046 | 5 | 43134968 | T | C | - | - | - | -2.03e-02 | 6.03e-02 | -1.83e-03 | 2.34e-02 | -1.73e-02 | 6.00e-02 | 6.93e-04 | 2.94e-02 | -4.84e-03 | 7.59e-02 | 3.99e-02 | 4.75e-02 |
| rs813301 | 5 | 52909927 | T | C | - | - | - | 1.48e-03 | 5.72e-02 | 3.29e-02 | 2.24e-02 | 1.11e-02 | 5.59e-02 | -4.63e-02 | 2.78e-02 | 1.32e-02 | 7.17e-02 | 4.44e-02 | 4.51e-02 |
| rs256350 | 5 | 59140876 | T | C | - | - | - | -6.41e-02 | 6.25e-02 | -1.07e-02 | 2.43e-02 | 4.62e-02 | 6.24e-02 | -2.59e-02 | 3.05e-02 | -1.57e-01 | 7.97e-02 | -1.70e-02 | 4.89e-02 |
| rs13173441 | 5 | 77048448 | T | C | rs1422409 | - | rs1422409 | 3.15e-02 | 8.75e-02 | -7.50e-02 | 3.26e-02 | 2.35e-02 | 9.10e-02 | 5.73e-02 | 4.31e-02 | 9.46e-02 | 1.11e-01 | 1.21e-01 | 7.10e-02 |
| rs17085593 | 5 | 95630705 | C | G | - | - | - | -1.87e-02 | 5.99e-02 | 2.23e-02 | 2.34e-02 | -3.71e-02 | 5.97e-02 | 2.84e-02 | 2.95e-02 | -6.01e-02 | 7.90e-02 | -1.64e-02 | 4.67e-02 |
| rs2916578 | 5 | 107316227 | A | G | rs288194 | - | rs288194 | 6.04e-03 | 5.76e-02 | -6.42e-03 | 2.17e-02 | 6.93e-02 | 5.52e-02 | 1.86e-03 | 2.73e-02 | 6.82e-02 | 7.40e-02 | 2.07e-02 | 4.38e-02 |
| rs654354 | 5 | 110503301 | A | T | - | - | - | 8.79e-03 | 6.08e-02 | -2.33e-02 | 2.23e-02 | -3.92e-02 | 5.97e-02 | -1.92e-02 | 2.80e-02 | -4.75e-02 | 8.00e-02 | -3.20e-02 | 4.48e-02 |
| rs247520 | 5 | 110876057 | T | C | - | - | - | 1.67e-01 | 6.64e-02 | -4.86e-03 | 2.58e-02 | 1.18e-01 | 6.36e-02 | 2.62e-02 | 3.27e-02 | 2.24e-02 | 8.11e-02 | 5.35e-02 | 5.28e-02 |
| rs1566385 | 5 | 111130474 | A | G | - | - | - | 2.69e-01 | 1.39e-01 | -6.33e-02 | 4.37e-02 | 6.34e-02 | 1.27e-01 | 1.14e-01 | 5.94e-02 | 2.06e-01 | 1.69e-01 | -8.58e-02 | 8.71e-02 |
| rs62379978 | 5 | 133915969 | T | G | - | - | - | -5.19e-02 | 8.22e-02 | -4.73e-02 | 3.01e-02 | -6.62e-02 | 7.82e-02 | 3.83e-02 | 3.90e-02 | 3.12e-02 | 1.05e-01 | 8.57e-03 | 6.17e-02 |
| rs3815212 | 5 | 137761555 | T | C | - | - | - | 6.34e-02 | 6.61e-02 | -1.02e-02 | 2.63e-02 | 6.45e-02 | 6.54e-02 | -1.00e-01 | 3.23e-02 | -6.84e-02 | 8.18e-02 | -3.38e-02 | 5.26e-02 |
| rs975642 | 5 | 139384490 | T | C | - | - | - | -1.70e-02 | 5.67e-02 | 1.34e-02 | 2.15e-02 | 5.08e-02 | 5.44e-02 | -2.16e-02 | 2.70e-02 | 1.27e-01 | 7.27e-02 | 1.95e-02 | 4.33e-02 |
| rs1428120 | 5 | 153541904 | T | G | - | - | - | -2.77e-02 | 5.60e-02 | -2.73e-02 | 2.16e-02 | -4.87e-02 | 5.51e-02 | 6.20e-03 | 2.73e-02 | 1.05e-01 | 7.14e-02 | -1.01e-01 | 4.34e-02 |
| rs437836 | 5 | 156715068 | T | C | - | - | - | 3.90e-03 | 7.33e-02 | 1.73e-02 | 2.90e-02 | 1.57e-02 | 7.32e-02 | 5.74e-03 | 3.65e-02 | -1.81e-01 | 1.00e-01 | -4.51e-02 | 5.95e-02 |
| rs9647570 | 5 | 167370263 | T | G | - | - | - | -1.42e-02 | 8.35e-02 | 1.06e-02 | 3.09e-02 | 2.43e-02 | 8.16e-02 | 4.56e-02 | 3.96e-02 | -9.75e-02 | 1.02e-01 | -4.93e-02 | 6.10e-02 |
| rs2546959 | 5 | 167404411 | T | G | - | - | - | -4.91e-02 | 7.69e-02 | -2.72e-02 | 2.91e-02 | 2.21e-02 | 7.24e-02 | -4.51e-02 | 3.63e-02 | 4.21e-02 | 9.43e-02 | 5.13e-02 | 6.01e-02 |
| rs4976623 | 5 | 167947996 | C | G | - | - | - | 9.03e-03 | 7.09e-02 | -2.36e-02 | 2.79e-02 | -4.92e-03 | 6.92e-02 | 5.16e-02 | 3.43e-02 | -1.54e-01 | 9.19e-02 | 1.06e-01 | 5.40e-02 |
| rs6864818 | 5 | 168734867 | T | C | - | - | - | -1.07e-01 | 6.80e-02 | -6.87e-03 | 2.69e-02 | -4.08e-02 | 6.72e-02 | -5.57e-05 | 3.38e-02 | -6.72e-02 | 8.77e-02 | -3.51e-02 | 5.47e-02 |
| rs4701140 | 5 | 179034260 | A | G | - | - | - | 1.31e-01 | 5.66e-02 | 4.15e-03 | 2.15e-02 | 7.39e-02 | 5.70e-02 | 5.28e-03 | 2.70e-02 | 1.29e-01 | 7.39e-02 | -2.23e-02 | 4.32e-02 |
| rs446745 | 6 | 14918298 | T | C | - | - | - | -3.23e-02 | 6.81e-02 | -5.43e-03 | 2.51e-02 | -1.20e-01 | 6.99e-02 | -3.19e-02 | 3.18e-02 | -6.70e-02 | 8.99e-02 | -1.99e-02 | 5.07e-02 |
| rs6927679 | 6 | 18559687 | T | C | - | - | - | 1.18e-01 | 6.45e-02 | 2.78e-02 | 2.38e-02 | 1.15e-01 | 6.14e-02 | 2.43e-02 | 2.99e-02 | 8.56e-02 | 7.99e-02 | 5.13e-02 | 4.82e-02 |
| rs1539310 | 6 | 22562485 | A | G | - | - | - | 2.71e-02 | 6.80e-02 | 1.29e-02 | 2.54e-02 | -2.27e-02 | 6.70e-02 | -2.78e-02 | 3.17e-02 | -1.15e-01 | 8.44e-02 | 9.31e-04 | 5.10e-02 |
| rs12663002 | 6 | 28441634 | T | C | - | - | - | 5.16e-02 | 8.39e-02 | -2.57e-02 | 3.27e-02 | 1.04e-02 | 8.05e-02 | -2.72e-02 | 4.12e-02 | -5.03e-02 | 1.07e-01 | -1.01e-01 | 6.78e-02 |
| rs62391851 | 6 | 29740548 | A | G | - | - | - | -1.24e-01 | 1.48e-01 | -5.05e-02 | 4.54e-02 | -1.67e-01 | 1.34e-01 | 5.76e-02 | 5.50e-02 | -3.06e-01 | 1.74e-01 | 5.86e-02 | 8.71e-02 |
| rs3021057 | 6 | 32652363 | T | C | - | - | - | -1.87e-02 | 5.89e-02 | -7.73e-04 | 2.16e-02 | -3.29e-02 | 5.59e-02 | -1.14e-02 | 2.71e-02 | -3.28e-03 | 7.24e-02 | 5.08e-02 | 4.34e-02 |
| rs9349203 | 6 | 41893323 | A | G | - | - | - | 5.88e-02 | 5.76e-02 | 6.96e-03 | 2.16e-02 | 1.46e-02 | 5.43e-02 | -1.60e-02 | 2.71e-02 | 5.25e-02 | 7.14e-02 | 1.71e-02 | 4.35e-02 |
| rs79541760 | 6 | 50930848 | A | T | - | - | - | -7.05e-03 | 7.64e-02 | -2.54e-02 | 2.83e-02 | 1.68e-02 | 7.36e-02 | 1.16e-02 | 3.61e-02 | 4.74e-02 | 9.96e-02 | -5.89e-02 | 5.64e-02 |
| rs222440 | 6 | 52946320 | T | C | - | - | - | 4.31e-02 | 7.35e-02 | -6.84e-03 | 2.81e-02 | 2.74e-02 | 7.22e-02 | -1.57e-03 | 3.53e-02 | -3.19e-03 | 9.47e-02 | -2.03e-02 | 5.68e-02 |
| rs9474996 | 6 | 54640512 | A | T | rs9474997 | - | rs9474997 | -4.98e-02 | 5.76e-02 | 4.91e-02 | 2.18e-02 | 1.25e-02 | 5.49e-02 | -8.24e-04 | 2.73e-02 | -9.74e-02 | 7.30e-02 | 3.26e-02 | 4.38e-02 |
| rs9382676 | 6 | 56859084 | T | C | - | - | - | -5.19e-02 | 7.20e-02 | -1.15e-03 | 2.59e-02 | 3.23e-02 | 6.90e-02 | -8.61e-04 | 3.25e-02 | 3.82e-03 | 8.77e-02 | 3.30e-03 | 5.21e-02 |
| rs7753896 | 6 | 76347020 | A | G | - | - | - | -2.79e-03 | 5.83e-02 | -1.34e-02 | 2.23e-02 | 5.91e-02 | 5.65e-02 | -4.05e-04 | 2.80e-02 | 9.95e-02 | 7.32e-02 | -7.12e-02 | 4.53e-02 |
| rs7757654 | 6 | 77173780 | T | C | - | - | - | 1.08e-01 | 6.43e-02 | -5.27e-03 | 2.32e-02 | 5.40e-02 | 6.04e-02 | 5.16e-02 | 2.89e-02 | -8.18e-03 | 7.93e-02 | 2.25e-02 | 4.65e-02 |
| rs1414186 | 6 | 77713859 | T | G | - | - | - | -3.30e-02 | 6.80e-02 | 2.83e-02 | 2.60e-02 | -8.18e-03 | 6.58e-02 | 1.85e-03 | 3.30e-02 | -3.78e-02 | 8.68e-02 | -3.17e-02 | 5.34e-02 |
| rs11756746 | 6 | 84286477 | A | G | - | - | - | -8.10e-02 | 6.39e-02 | 1.56e-02 | 2.48e-02 | -1.27e-02 | 6.31e-02 | -1.82e-02 | 3.15e-02 | -5.59e-02 | 8.50e-02 | 1.79e-02 | 5.00e-02 |
| rs6931884 | 6 | 100158873 | T | C | - | - | - | -1.57e-01 | 8.65e-02 | 2.14e-02 | 3.10e-02 | -8.73e-02 | 8.25e-02 | -3.28e-02 | 3.97e-02 | -8.70e-02 | 1.11e-01 | -9.18e-02 | 6.51e-02 |
| rs9403051 | 6 | 100194846 | A | G | - | - | - | -6.73e-02 | 5.77e-02 | -7.41e-03 | 2.17e-02 | -8.34e-02 | 5.78e-02 | -4.10e-02 | 2.73e-02 | 2.03e-02 | 7.21e-02 | 1.70e-02 | 4.38e-02 |
| rs13199764 | 6 | 100744134 | T | C | - | - | - | 5.98e-02 | 6.78e-02 | -5.18e-02 | 2.55e-02 | 1.29e-01 | 6.68e-02 | 6.67e-02 | 3.32e-02 | 2.77e-02 | 8.53e-02 | 4.67e-02 | 5.26e-02 |
| rs12200565 | 6 | 100983589 | T | C | - | - | - | -3.97e-02 | 5.87e-02 | 1.44e-03 | 2.14e-02 | 1.21e-02 | 5.60e-02 | 1.64e-02 | 2.70e-02 | 9.99e-02 | 7.30e-02 | -3.06e-02 | 4.32e-02 |
| rs395962 | 6 | 105397418 | T | G | - | - | - | -9.10e-03 | 6.02e-02 | -4.00e-02 | 2.32e-02 | 3.86e-02 | 5.70e-02 | -3.27e-02 | 2.92e-02 | 6.85e-02 | 7.57e-02 | 2.93e-02 | 4.61e-02 |
| rs6911407 | 6 | 108867031 | A | C | - | - | - | -4.26e-02 | 5.94e-02 | -2.19e-02 | 2.22e-02 | 5.52e-03 | 5.78e-02 | 2.84e-02 | 2.77e-02 | -5.65e-02 | 7.75e-02 | -9.04e-03 | 4.46e-02 |
| rs235696 | 6 | 124253495 | C | G | rs53359 | - | rs53359 | 3.20e-02 | 5.86e-02 | 2.66e-03 | 2.26e-02 | -7.35e-02 | 5.68e-02 | -3.37e-02 | 2.83e-02 | -1.15e-02 | 7.52e-02 | -4.43e-02 | 4.53e-02 |
| rs4897178 | 6 | 126727908 | T | G | rs9398805 | - | rs9398805 | 1.40e-02 | 5.63e-02 | 3.33e-02 | 2.16e-02 | 3.81e-02 | 5.39e-02 | -6.43e-02 | 2.71e-02 | 4.79e-02 | 7.27e-02 | -6.09e-02 | 4.34e-02 |
| rs4327718 | 6 | 128364709 | A | G | - | - | - | 8.35e-02 | 7.81e-02 | 2.14e-02 | 2.80e-02 | 1.67e-01 | 7.80e-02 | -1.92e-02 | 3.46e-02 | 2.67e-02 | 9.63e-02 | -5.41e-03 | 5.59e-02 |
| rs78928932 | 6 | 136228617 | T | C | - | - | - | -8.36e-02 | 1.07e-01 | -1.06e-01 | 4.11e-02 | -1.05e-01 | 1.09e-01 | 1.12e-01 | 5.62e-02 | -1.16e-01 | 1.36e-01 | 2.54e-01 | 9.59e-02 |
| rs117530880 | 6 | 146687748 | T | G | - | - | - | 3.22e-01 | 1.57e-01 | -9.34e-02 | 5.95e-02 | 2.50e-01 | 1.50e-01 | -4.22e-02 | 7.66e-02 | 8.94e-02 | 2.08e-01 | -2.47e-02 | 1.23e-01 |
| rs6911527 | 6 | 148285329 | T | C | - | - | - | -3.27e-03 | 6.71e-02 | 1.31e-02 | 2.54e-02 | -3.51e-02 | 6.73e-02 | -2.92e-02 | 3.24e-02 | -9.68e-02 | 9.26e-02 | 9.93e-03 | 5.14e-02 |
| rs6933660 | 6 | 151803754 | A | C | - | - | - | -8.44e-02 | 6.03e-02 | -4.38e-03 | 2.31e-02 | -5.80e-02 | 6.10e-02 | -1.45e-02 | 2.92e-02 | -1.29e-01 | 7.77e-02 | 9.84e-02 | 4.58e-02 |
| rs910425 | 6 | 170652191 | A | G | - | - | - | -7.88e-02 | 5.74e-02 | 9.18e-03 | 2.16e-02 | 9.10e-02 | 5.71e-02 | 2.25e-02 | 2.71e-02 | 1.59e-02 | 7.47e-02 | 2.80e-02 | 4.35e-02 |
| rs10268051 | 7 | 27763590 | A | C | - | - | - | -5.42e-02 | 6.61e-02 | -1.62e-02 | 2.58e-02 | 5.25e-02 | 6.42e-02 | -3.63e-02 | 3.23e-02 | 2.36e-02 | 8.54e-02 | 4.95e-02 | 5.30e-02 |
| rs17171852 | 7 | 41392815 | A | C | - | - | - | 1.11e-01 | 7.09e-02 | -3.34e-02 | 2.66e-02 | 7.36e-02 | 6.83e-02 | -8.22e-02 | 3.30e-02 | 1.91e-01 | 9.52e-02 | 1.05e-01 | 5.59e-02 |
| rs1079866 | 7 | 41470093 | C | G | - | - | - | 2.77e-02 | 8.74e-02 | -2.23e-02 | 3.11e-02 | -8.87e-02 | 8.59e-02 | -2.31e-02 | 3.91e-02 | -2.26e-01 | 1.03e-01 | -7.04e-02 | 6.16e-02 |
| rs1470750 | 7 | 50576648 | C | G | - | - | - | 7.89e-02 | 5.47e-02 | 3.55e-02 | 2.20e-02 | 4.43e-02 | 5.36e-02 | -5.05e-04 | 2.77e-02 | 9.98e-02 | 6.79e-02 | -4.58e-02 | 4.40e-02 |
| rs2267812 | 7 | 74138121 | A | C | - | - | - | 3.03e-02 | 7.34e-02 | -3.89e-02 | 2.62e-02 | 1.23e-02 | 7.16e-02 | 1.45e-02 | 3.35e-02 | -9.27e-02 | 9.10e-02 | -7.58e-02 | 5.23e-02 |
| rs1030015 | 7 | 78139581 | T | G | - | - | - | 6.56e-02 | 5.60e-02 | 8.73e-03 | 2.15e-02 | 4.34e-02 | 5.49e-02 | 1.03e-02 | 2.70e-02 | 4.67e-02 | 7.30e-02 | -4.26e-02 | 4.32e-02 |
| rs149226155 | 7 | 93215658 | A | G | rs13247665 | - | rs13247665 | 5.11e-02 | 5.70e-02 | -1.83e-02 | 2.25e-02 | -4.37e-02 | 5.61e-02 | -5.17e-02 | 2.85e-02 | -4.68e-03 | 7.56e-02 | -3.86e-02 | 4.54e-02 |
| rs15671 | 7 | 94186064 | A | C | - | - | - | -8.81e-02 | 5.87e-02 | 6.13e-03 | 2.18e-02 | -2.09e-02 | 5.70e-02 | -1.32e-02 | 2.74e-02 | 7.89e-02 | 7.51e-02 | 4.47e-02 | 4.37e-02 |
| rs999885 | 7 | 99701176 | A | G | - | - | - | -3.16e-02 | 5.71e-02 | 1.14e-02 | 2.15e-02 | -3.62e-02 | 5.47e-02 | 1.41e-02 | 2.71e-02 | 1.27e-01 | 7.76e-02 | 4.76e-02 | 4.34e-02 |
| rs1456031 | 7 | 114296102 | T | C | - | - | - | -7.31e-02 | 5.56e-02 | -1.64e-02 | 2.17e-02 | -4.65e-02 | 5.45e-02 | -2.34e-02 | 2.73e-02 | -1.42e-01 | 7.37e-02 | -5.03e-03 | 4.36e-02 |
| rs10237306 | 7 | 121955981 | T | G | - | - | - | 3.29e-02 | 5.93e-02 | 1.37e-02 | 2.20e-02 | 2.13e-03 | 6.04e-02 | -1.58e-02 | 2.77e-02 | 8.03e-03 | 7.58e-02 | 2.06e-02 | 4.42e-02 |
| rs11767400 | 7 | 122160742 | A | C | - | - | - | 4.89e-04 | 5.90e-02 | -1.94e-02 | 2.37e-02 | -9.74e-02 | 5.94e-02 | 3.03e-02 | 2.95e-02 | -1.01e-01 | 7.88e-02 | -6.20e-02 | 4.81e-02 |
| rs11556924 | 7 | 129663496 | T | C | - | - | - | 7.55e-02 | 5.99e-02 | -3.28e-02 | 2.23e-02 | 9.53e-02 | 6.16e-02 | -8.83e-03 | 2.80e-02 | 1.78e-02 | 7.57e-02 | 6.70e-02 | 4.44e-02 |
| rs17563472 | 7 | 130409054 | T | C | - | - | - | 2.38e-01 | 1.64e-01 | -8.15e-02 | 5.45e-02 | 5.03e-03 | 1.48e-01 | 8.24e-02 | 7.41e-02 | 4.22e-01 | 2.29e-01 | 4.81e-02 | 1.17e-01 |
| rs12707076 | 7 | 132729814 | C | G | - | - | - | 2.73e-02 | 5.70e-02 | 1.61e-02 | 2.21e-02 | -2.76e-02 | 5.63e-02 | 3.52e-03 | 2.79e-02 | -5.44e-02 | 7.38e-02 | -4.14e-02 | 4.49e-02 |
| rs13233916 | 7 | 138874416 | C | G | - | - | - | 1.59e-02 | 1.06e-01 | -6.65e-02 | 3.66e-02 | 2.01e-02 | 1.09e-01 | -1.51e-03 | 4.72e-02 | 2.31e-02 | 1.31e-01 | 3.72e-02 | 7.65e-02 |
| rs7004265 | 8 | 1523903 | T | C | rs12546094 | - | rs12546094 | 3.80e-02 | 5.60e-02 | -5.92e-03 | 2.16e-02 | 9.93e-02 | 5.43e-02 | -8.87e-03 | 2.73e-02 | 8.28e-02 | 7.37e-02 | 4.24e-02 | 4.35e-02 |
| rs2688326 | 8 | 3767623 | T | C | - | - | - | 1.11e-01 | 6.23e-02 | 2.31e-04 | 2.34e-02 | 9.13e-02 | 6.00e-02 | -1.09e-02 | 2.94e-02 | -2.18e-02 | 8.01e-02 | 1.33e-02 | 4.72e-02 |
| rs2724961 | 8 | 4560227 | T | C | - | - | - | -3.49e-02 | 5.60e-02 | -1.48e-02 | 2.17e-02 | 6.43e-03 | 5.41e-02 | -1.53e-02 | 2.73e-02 | -1.80e-03 | 7.04e-02 | -7.83e-02 | 4.38e-02 |
| rs4875424 | 8 | 4831685 | T | C | - | - | - | -8.34e-02 | 6.00e-02 | 1.44e-02 | 2.24e-02 | -2.65e-02 | 5.69e-02 | -7.16e-03 | 2.83e-02 | -1.03e-01 | 7.38e-02 | 5.26e-02 | 4.49e-02 |
| rs6185 | 8 | 25280800 | C | G | - | - | - | 4.06e-02 | 6.34e-02 | 7.41e-02 | 2.55e-02 | 9.11e-02 | 6.36e-02 | 1.06e-02 | 3.15e-02 | 1.11e-01 | 8.11e-02 | -1.68e-03 | 5.02e-02 |
| rs13278754 | 8 | 34902952 | C | G | - | - | - | -2.30e-02 | 6.21e-02 | 5.02e-02 | 2.37e-02 | -1.24e-03 | 6.11e-02 | -1.19e-02 | 3.02e-02 | 7.12e-02 | 7.99e-02 | 5.75e-02 | 4.76e-02 |
| rs4487799 | 8 | 53163528 | A | T | - | - | - | 1.13e-02 | 5.98e-02 | -1.98e-02 | 2.37e-02 | 1.15e-02 | 6.31e-02 | 2.32e-02 | 2.94e-02 | 1.45e-03 | 7.56e-02 | 9.20e-02 | 4.66e-02 |
| rs16918378 | 8 | 53877882 | T | C | - | - | - | -1.78e-02 | 8.26e-02 | -5.56e-04 | 3.30e-02 | -1.10e-01 | 8.11e-02 | -3.91e-02 | 4.08e-02 | 3.50e-03 | 1.08e-01 | 9.23e-03 | 6.69e-02 |
| rs56409371 | 8 | 53934144 | A | G | - | - | - | -1.40e-02 | 6.91e-02 | -1.90e-02 | 2.63e-02 | 2.65e-02 | 6.71e-02 | 2.25e-02 | 3.36e-02 | -9.72e-02 | 8.65e-02 | 3.30e-02 | 5.39e-02 |
| rs1449543 | 8 | 76591987 | T | C | - | - | - | -2.08e-02 | 5.70e-02 | 1.05e-03 | 2.15e-02 | -6.13e-02 | 5.59e-02 | -2.00e-02 | 2.71e-02 | -2.38e-02 | 7.41e-02 | 3.48e-02 | 4.33e-02 |
| rs11786868 | 8 | 77653945 | C | G | - | - | - | 5.14e-02 | 7.86e-02 | -1.43e-02 | 2.91e-02 | 4.19e-02 | 8.01e-02 | -4.12e-02 | 3.63e-02 | 6.20e-02 | 1.06e-01 | -9.87e-02 | 5.69e-02 |
| rs10094506 | 8 | 78116203 | T | C | rs4735761 | - | rs4735761 | -4.35e-02 | 6.07e-02 | 9.13e-03 | 2.38e-02 | -8.14e-03 | 6.05e-02 | -1.22e-02 | 3.02e-02 | -6.87e-03 | 7.81e-02 | 3.87e-02 | 4.77e-02 |
| rs35485457 | 8 | 78679087 | T | G | - | - | - | -5.85e-02 | 6.48e-02 | -3.43e-02 | 2.32e-02 | 1.52e-02 | 6.38e-02 | -1.48e-02 | 2.91e-02 | -1.15e-01 | 8.59e-02 | 2.95e-02 | 4.61e-02 |
| rs2441873 | 8 | 105329549 | T | G | rs2514653 | - | rs2514653 | -1.69e-01 | 6.09e-02 | -2.11e-02 | 2.20e-02 | -7.56e-02 | 5.81e-02 | -1.66e-02 | 2.78e-02 | -2.43e-02 | 7.43e-02 | 9.34e-02 | 4.40e-02 |
| rs7826872 | 8 | 132071766 | T | C | - | - | - | 4.71e-02 | 5.63e-02 | -2.66e-03 | 2.16e-02 | 2.58e-02 | 5.67e-02 | 2.06e-02 | 2.71e-02 | -4.83e-02 | 7.09e-02 | 1.50e-02 | 4.35e-02 |
| rs552491 | 9 | 1711210 | A | G | - | - | - | -6.72e-02 | 6.00e-02 | 2.90e-03 | 2.22e-02 | -3.53e-02 | 5.71e-02 | 3.88e-02 | 2.81e-02 | 7.88e-02 | 7.58e-02 | 2.23e-02 | 4.49e-02 |
| rs913588 | 9 | 7174673 | A | G | - | - | - | -5.82e-02 | 5.66e-02 | 6.39e-03 | 2.15e-02 | -2.60e-02 | 5.41e-02 | -1.78e-02 | 2.70e-02 | -6.81e-02 | 7.34e-02 | 1.28e-02 | 4.33e-02 |
| rs10959016 | 9 | 10283451 | A | G | - | - | - | -6.43e-02 | 7.26e-02 | 3.87e-02 | 2.59e-02 | -1.76e-02 | 7.05e-02 | 3.34e-02 | 3.27e-02 | -4.76e-02 | 9.49e-02 | 2.00e-02 | 5.26e-02 |
| rs10959552 | 9 | 11130009 | A | G | - | - | - | 1.01e-01 | 8.66e-02 | 2.78e-02 | 3.32e-02 | 9.52e-02 | 8.37e-02 | -3.07e-02 | 4.09e-02 | 4.38e-02 | 1.12e-01 | 2.42e-02 | 6.66e-02 |
| rs1601615 | 9 | 11813745 | T | C | - | - | - | 5.53e-03 | 5.68e-02 | 2.27e-02 | 2.19e-02 | -1.34e-02 | 5.61e-02 | 4.56e-02 | 2.75e-02 | 8.34e-02 | 7.49e-02 | -5.44e-02 | 4.46e-02 |
| rs7849973 | 9 | 22819576 | C | G | - | - | - | 2.61e-02 | 5.73e-02 | 1.30e-02 | 2.29e-02 | 4.85e-02 | 5.53e-02 | -1.29e-02 | 2.87e-02 | -6.36e-02 | 7.20e-02 | 4.46e-02 | 4.64e-02 |
| rs1329767 | 9 | 73798371 | A | C | - | - | - | -1.30e-02 | 5.97e-02 | 1.55e-02 | 2.23e-02 | 5.40e-02 | 5.83e-02 | 1.68e-03 | 2.81e-02 | 4.00e-02 | 7.63e-02 | 4.32e-02 | 4.48e-02 |
| rs2604265 | 9 | 76905178 | A | G | - | - | - | -9.98e-02 | 6.14e-02 | -2.02e-02 | 2.46e-02 | 7.97e-02 | 6.21e-02 | 1.50e-02 | 3.07e-02 | -6.86e-02 | 7.82e-02 | 9.14e-02 | 4.83e-02 |
| rs35436838 | 9 | 77273910 | T | G | - | - | - | -3.70e-02 | 1.62e-01 | -2.81e-02 | 4.97e-02 | 6.09e-02 | 1.62e-01 | 7.39e-02 | 6.49e-02 | -3.96e-01 | 1.83e-01 | -1.62e-01 | 9.49e-02 |
| rs2378100 | 9 | 80513323 | T | C | - | - | - | 5.00e-02 | 5.86e-02 | 2.62e-02 | 2.17e-02 | 9.04e-02 | 5.82e-02 | 3.40e-02 | 2.74e-02 | -7.91e-02 | 7.36e-02 | -3.44e-02 | 4.37e-02 |
| rs4877387 | 9 | 81679875 | T | C | - | - | - | -7.17e-03 | 6.48e-02 | 6.67e-03 | 2.37e-02 | -4.99e-02 | 6.58e-02 | 3.04e-02 | 2.97e-02 | 2.34e-03 | 8.53e-02 | 9.22e-02 | 4.70e-02 |
| rs11534296 | 9 | 83282402 | A | G | - | - | - | -1.85e-01 | 6.40e-02 | 1.74e-02 | 2.42e-02 | -6.33e-02 | 6.05e-02 | 4.69e-02 | 3.02e-02 | -9.43e-02 | 8.04e-02 | -4.96e-02 | 4.94e-02 |
| rs7853970 | 9 | 86715566 | T | C | - | - | - | -3.45e-02 | 5.86e-02 | 1.93e-03 | 2.18e-02 | -2.59e-02 | 5.74e-02 | -6.98e-03 | 2.75e-02 | 5.20e-02 | 7.41e-02 | 1.56e-02 | 4.39e-02 |
| rs13283567 | 9 | 86764996 | T | C | - | - | - | 8.15e-02 | 7.89e-02 | 5.10e-02 | 2.96e-02 | 5.40e-02 | 7.80e-02 | 6.29e-02 | 3.71e-02 | 3.62e-02 | 1.03e-01 | -1.64e-02 | 6.10e-02 |
| rs1571536 | 9 | 92215638 | T | C | - | - | - | 1.81e-01 | 5.66e-02 | -2.22e-03 | 2.16e-02 | 1.09e-01 | 5.50e-02 | -4.24e-04 | 2.72e-02 | 2.24e-01 | 7.03e-02 | -2.80e-02 | 4.35e-02 |
| rs9330454 | 9 | 92515514 | A | G | - | - | - | -5.80e-02 | 5.72e-02 | -1.12e-02 | 2.18e-02 | -4.78e-02 | 5.62e-02 | -7.47e-02 | 2.76e-02 | -1.35e-01 | 7.17e-02 | -1.18e-02 | 4.40e-02 |
| rs10992769 | 9 | 96276910 | C | G | - | - | - | -3.67e-02 | 6.18e-02 | -1.11e-02 | 2.39e-02 | -3.87e-02 | 6.01e-02 | 5.83e-02 | 3.05e-02 | 4.12e-02 | 8.13e-02 | -3.53e-02 | 4.79e-02 |
| rs10156597 | 9 | 108941509 | A | T | - | - | - | -5.39e-02 | 6.15e-02 | 2.58e-02 | 2.33e-02 | -5.42e-02 | 5.81e-02 | 1.47e-03 | 2.91e-02 | 7.65e-02 | 7.79e-02 | -5.59e-02 | 4.61e-02 |
| rs56927240 | 9 | 109148074 | T | C | rs2417690 | - | rs2417690 | 2.29e-03 | 6.66e-02 | 4.61e-02 | 1.00e-01 | -3.06e-02 | 6.79e-02 | -1.32e-01 | 1.33e-01 | -1.16e-01 | 8.70e-02 | 1.28e-02 | 2.02e-01 |
| rs10978641 | 9 | 109554196 | A | T | - | - | - | 1.24e-01 | 6.55e-02 | 4.01e-02 | 2.48e-02 | 7.29e-02 | 6.53e-02 | -2.01e-02 | 3.07e-02 | 1.60e-01 | 8.51e-02 | 4.14e-02 | 5.00e-02 |
| rs11792861 | 9 | 111809295 | A | C | - | - | - | -4.90e-02 | 6.16e-02 | 5.56e-03 | 2.38e-02 | 5.10e-02 | 6.12e-02 | -6.07e-02 | 2.96e-02 | -2.18e-03 | 8.12e-02 | -5.64e-02 | 4.73e-02 |
| rs7852169 | 9 | 114318394 | C | G | - | - | - | -4.28e-02 | 9.38e-02 | 4.06e-02 | 3.88e-02 | -4.72e-02 | 9.49e-02 | 2.17e-02 | 4.83e-02 | 5.27e-02 | 1.20e-01 | -9.36e-02 | 7.42e-02 |
| rs4836984 | 9 | 127405632 | T | C | - | - | - | 1.06e-01 | 5.61e-02 | -2.86e-02 | 2.14e-02 | 6.33e-02 | 5.59e-02 | 3.91e-02 | 2.70e-02 | 1.58e-01 | 7.22e-02 | 4.50e-02 | 4.32e-02 |
| rs467379 | 9 | 136905474 | T | C | - | - | - | 4.30e-02 | 6.08e-02 | 1.75e-02 | 2.37e-02 | 6.19e-02 | 5.93e-02 | -2.60e-02 | 3.01e-02 | 2.18e-02 | 8.05e-02 | -1.61e-02 | 4.80e-02 |
| rs7907759 | 10 | 1730008 | A | G | - | - | - | -9.24e-04 | 5.54e-02 | 1.69e-02 | 2.16e-02 | 1.15e-02 | 5.59e-02 | -2.78e-02 | 2.72e-02 | -1.19e-02 | 7.77e-02 | 1.94e-02 | 4.35e-02 |
| rs7912468 | 10 | 2697434 | T | C | - | - | - | -4.75e-02 | 5.73e-02 | -1.10e-02 | 2.18e-02 | -3.96e-02 | 5.68e-02 | -4.26e-02 | 2.74e-02 | 5.29e-02 | 7.26e-02 | 7.98e-02 | 4.42e-02 |
| rs1885740 | 10 | 10251910 | A | G | - | - | - | 3.49e-02 | 6.26e-02 | 2.62e-02 | 2.40e-02 | 1.38e-02 | 6.27e-02 | 5.25e-02 | 3.01e-02 | 7.50e-03 | 8.16e-02 | -2.58e-02 | 4.90e-02 |
| rs10906395 | 10 | 13541008 | T | C | - | - | - | -3.89e-02 | 5.79e-02 | 4.72e-02 | 2.20e-02 | 7.88e-02 | 5.60e-02 | -5.68e-02 | 2.74e-02 | 1.03e-01 | 7.52e-02 | 6.83e-02 | 4.45e-02 |
| rs61846901 | 10 | 51056858 | T | C | - | - | - | 1.04e-01 | 6.00e-02 | -4.27e-02 | 2.33e-02 | 5.32e-02 | 5.92e-02 | -2.92e-02 | 2.93e-02 | 6.96e-02 | 7.84e-02 | -8.18e-02 | 4.74e-02 |
| rs6415872 | 10 | 63660689 | A | G | - | - | - | -4.52e-02 | 5.63e-02 | 5.48e-03 | 2.15e-02 | 8.16e-03 | 5.64e-02 | 1.61e-02 | 2.70e-02 | -7.71e-02 | 7.45e-02 | 9.19e-03 | 4.33e-02 |
| rs4746113 | 10 | 74071178 | A | G | - | - | - | 1.59e-02 | 6.42e-02 | -4.84e-02 | 2.36e-02 | 3.12e-02 | 6.24e-02 | -2.53e-02 | 2.95e-02 | 1.08e-02 | 8.39e-02 | -9.40e-03 | 4.71e-02 |
| rs77532868 | 10 | 88081438 | T | C | - | - | - | -3.25e-01 | 1.46e-01 | 1.63e-02 | 4.80e-02 | -1.27e-01 | 1.33e-01 | -3.28e-02 | 6.17e-02 | -1.36e-01 | 1.67e-01 | 2.68e-02 | 9.59e-02 |
| rs1172955 | 10 | 97877320 | A | T | - | - | - | -6.07e-02 | 6.33e-02 | -8.02e-03 | 2.34e-02 | 1.23e-02 | 6.33e-02 | 2.60e-02 | 2.97e-02 | -2.12e-01 | 8.10e-02 | 1.15e-02 | 4.75e-02 |
| rs72842141 | 10 | 102686073 | A | T | - | - | - | -4.16e-03 | 1.09e-01 | -8.48e-03 | 4.33e-02 | 7.73e-02 | 1.16e-01 | 1.83e-02 | 5.50e-02 | 1.80e-01 | 1.47e-01 | -2.87e-02 | 8.63e-02 |
| rs59543819 | 10 | 103754188 | T | C | - | - | - | 9.02e-03 | 6.10e-02 | 1.91e-02 | 2.37e-02 | -4.31e-03 | 6.51e-02 | 2.90e-02 | 2.99e-02 | 5.22e-02 | 7.97e-02 | 7.46e-02 | 4.84e-02 |
| rs2066323 | 10 | 104871361 | A | G | - | - | - | 7.83e-02 | 5.58e-02 | -5.79e-03 | 2.19e-02 | -4.39e-02 | 5.40e-02 | -3.75e-02 | 2.75e-02 | -6.07e-02 | 7.13e-02 | -6.27e-02 | 4.39e-02 |
| rs10885077 | 10 | 112759731 | T | G | - | - | - | 1.40e-02 | 6.95e-02 | -3.18e-03 | 2.42e-02 | -9.84e-02 | 6.67e-02 | -1.59e-02 | 3.06e-02 | 4.21e-03 | 8.60e-02 | 7.60e-02 | 4.80e-02 |
| rs4751614 | 10 | 118696266 | A | T | - | - | - | 4.92e-02 | 6.62e-02 | -4.67e-03 | 2.54e-02 | -6.88e-02 | 6.17e-02 | 8.85e-03 | 3.21e-02 | -2.95e-02 | 8.12e-02 | 3.28e-03 | 5.12e-02 |
| rs10400136 | 10 | 120833948 | A | G | - | - | - | 7.97e-02 | 5.77e-02 | 2.02e-02 | 2.16e-02 | 7.22e-02 | 5.84e-02 | -4.09e-02 | 2.71e-02 | -6.61e-03 | 7.40e-02 | 1.01e-01 | 4.37e-02 |
| rs73435048 | 10 | 121154531 | A | G | - | - | - | 3.13e-02 | 1.18e-01 | -2.91e-03 | 4.45e-02 | -4.89e-02 | 1.22e-01 | 9.69e-02 | 5.39e-02 | -1.36e-01 | 1.68e-01 | -1.12e-01 | 9.41e-02 |
| rs12571664 | 10 | 121708929 | T | C | - | - | - | -2.16e-01 | 6.77e-02 | 1.41e-02 | 2.79e-02 | -1.16e-01 | 6.68e-02 | 3.74e-02 | 3.53e-02 | -1.84e-02 | 9.36e-02 | -2.69e-03 | 5.58e-02 |
| rs7077302 | 10 | 123676662 | C | G | - | - | - | -2.42e-02 | 1.00e-01 | 5.50e-02 | 3.82e-02 | -1.22e-01 | 1.01e-01 | 9.73e-03 | 4.90e-02 | -2.33e-01 | 1.34e-01 | 7.39e-02 | 7.67e-02 |
| rs4576738 | 10 | 134294398 | A | G | rs7904728 | rs4412685 | rs7904728 | 1.83e-02 | 5.62e-02 | -3.74e-02 | 2.16e-02 | 1.13e-02 | 5.40e-02 | 3.32e-02 | 2.71e-02 | 4.82e-02 | 7.06e-02 | 7.48e-03 | 4.34e-02 |
| rs3782120 | 11 | 206089 | A | G | - | - | - | 5.47e-02 | 6.38e-02 | -2.25e-02 | 2.51e-02 | 3.48e-02 | 6.22e-02 | -1.63e-03 | 3.14e-02 | -1.20e-01 | 8.40e-02 | 3.48e-02 | 4.98e-02 |
| rs16937956 | 11 | 8404501 | A | G | - | - | - | 2.83e-02 | 5.71e-02 | 5.00e-02 | 2.26e-02 | 7.01e-02 | 5.90e-02 | -5.44e-03 | 2.83e-02 | 9.07e-02 | 7.27e-02 | -3.29e-02 | 4.50e-02 |
| rs10832021 | 11 | 13324530 | A | G | - | - | - | -4.80e-02 | 6.40e-02 | 4.25e-02 | 2.37e-02 | 5.13e-02 | 6.60e-02 | 2.99e-02 | 2.98e-02 | 3.04e-03 | 8.10e-02 | 3.19e-02 | 4.77e-02 |
| rs4359170 | 11 | 16596152 | A | T | - | - | - | -3.81e-02 | 5.89e-02 | -1.36e-02 | 2.28e-02 | -5.20e-02 | 5.78e-02 | 1.84e-02 | 2.88e-02 | -1.44e-01 | 7.51e-02 | 6.64e-02 | 4.65e-02 |
| rs1032682 | 11 | 22791324 | T | C | - | - | - | -3.66e-02 | 5.79e-02 | 2.57e-02 | 2.18e-02 | -6.07e-02 | 5.62e-02 | 2.20e-02 | 2.75e-02 | -2.79e-02 | 7.43e-02 | -1.61e-02 | 4.41e-02 |
| rs16917237 | 11 | 27702383 | T | G | - | - | - | 5.51e-02 | 6.90e-02 | -1.38e-02 | 2.69e-02 | 1.35e-02 | 6.65e-02 | -3.85e-02 | 3.41e-02 | 1.02e-02 | 8.80e-02 | -7.68e-02 | 5.51e-02 |
| rs11606190 | 11 | 28033473 | A | G | - | - | - | -2.92e-02 | 8.29e-02 | -2.74e-02 | 3.04e-02 | -7.13e-02 | 7.93e-02 | 2.02e-02 | 3.78e-02 | -6.55e-02 | 1.06e-01 | 8.92e-02 | 5.90e-02 |
| rs6484408 | 11 | 28899164 | A | G | rs10742205 | - | rs10742205 | 6.83e-02 | 6.31e-02 | -1.77e-03 | 2.43e-02 | 5.65e-02 | 6.08e-02 | 2.13e-02 | 3.05e-02 | 2.11e-01 | 7.82e-02 | -1.78e-02 | 4.91e-02 |
| rs11031040 | 11 | 30317733 | T | G | - | - | - | 6.63e-03 | 7.13e-02 | 7.58e-02 | 3.01e-02 | 4.76e-03 | 7.20e-02 | 2.58e-02 | 3.72e-02 | 5.53e-03 | 8.99e-02 | -1.45e-01 | 5.64e-02 |
| rs1023955 | 11 | 43608835 | T | G | - | - | - | 3.57e-02 | 5.81e-02 | 4.83e-02 | 2.17e-02 | -2.14e-02 | 5.69e-02 | 1.42e-02 | 2.74e-02 | 2.04e-03 | 7.41e-02 | -9.14e-03 | 4.41e-02 |
| rs970179 | 11 | 45433845 | A | G | - | - | - | -1.13e-02 | 5.55e-02 | -4.14e-03 | 2.15e-02 | 5.43e-03 | 5.43e-02 | 1.83e-02 | 2.70e-02 | 6.97e-02 | 7.30e-02 | -4.04e-02 | 4.34e-02 |
| rs953230 | 11 | 46064974 | A | G | - | - | - | 5.09e-03 | 6.13e-02 | -7.54e-03 | 2.36e-02 | 2.32e-02 | 6.12e-02 | -8.57e-03 | 2.98e-02 | -6.38e-02 | 7.92e-02 | -1.07e-01 | 4.68e-02 |
| rs68002803 | 11 | 46539110 | T | C | - | - | - | -4.10e-02 | 6.07e-02 | -4.15e-03 | 2.31e-02 | -5.12e-02 | 5.93e-02 | 2.60e-02 | 2.94e-02 | 8.00e-02 | 7.90e-02 | -2.06e-02 | 4.65e-02 |
| rs10750766 | 11 | 65473798 | A | C | - | - | - | -6.67e-02 | 6.17e-02 | -2.27e-02 | 2.34e-02 | -1.86e-02 | 6.03e-02 | 2.82e-02 | 2.98e-02 | -9.73e-03 | 8.24e-02 | 6.47e-02 | 4.80e-02 |
| rs7115444 | 11 | 77555824 | T | C | - | - | - | 9.19e-02 | 7.16e-02 | 1.95e-02 | 2.66e-02 | 1.07e-01 | 7.07e-02 | 5.93e-02 | 3.29e-02 | 5.28e-02 | 9.37e-02 | -4.86e-02 | 5.45e-02 |
| rs4945266 | 11 | 78027488 | A | G | - | - | - | 7.63e-02 | 7.60e-02 | 4.10e-02 | 2.97e-02 | -1.18e-02 | 7.20e-02 | -2.36e-02 | 3.67e-02 | -1.48e-01 | 9.16e-02 | 4.41e-02 | 5.98e-02 |
| rs4402316 | 11 | 84780098 | C | G | rs11234273 | - | rs11234273 | 3.46e-02 | 6.49e-02 | 3.36e-02 | 2.42e-02 | 3.15e-02 | 6.43e-02 | 1.20e-02 | 3.06e-02 | -5.35e-02 | 8.37e-02 | -7.86e-02 | 5.01e-02 |
| rs7108556 | 11 | 86716236 | T | C | - | - | - | 1.49e-01 | 7.03e-02 | 1.49e-03 | 2.59e-02 | 1.32e-01 | 6.66e-02 | 4.88e-02 | 3.30e-02 | 5.23e-02 | 8.54e-02 | 1.81e-02 | 5.23e-02 |
| rs113557523 | 11 | 94085099 | T | C | rs57646575 | - | rs57646575 | -7.89e-02 | 9.39e-02 | 3.17e-05 | 3.95e-02 | -7.25e-02 | 9.41e-02 | 2.49e-02 | 4.94e-02 | -6.63e-02 | 1.24e-01 | -1.03e-01 | 8.32e-02 |
| rs6590889 | 11 | 101438191 | T | C | - | - | - | -3.65e-03 | 5.80e-02 | 3.07e-02 | 2.26e-02 | -9.49e-02 | 5.84e-02 | 6.47e-03 | 2.85e-02 | 3.60e-03 | 7.35e-02 | -1.98e-02 | 4.59e-02 |
| rs17564430 | 11 | 115043574 | T | G | - | - | - | -6.18e-02 | 6.22e-02 | 1.18e-02 | 2.46e-02 | -1.35e-01 | 6.07e-02 | -2.36e-02 | 3.08e-02 | 4.32e-03 | 8.38e-02 | -1.58e-02 | 4.93e-02 |
| rs77530428 | 12 | 17126283 | A | G | - | - | - | 4.06e-01 | 2.51e-01 | 1.69e-01 | 9.67e-02 | 5.12e-01 | 2.97e-01 | 4.41e-02 | 1.15e-01 | 4.15e-01 | 3.39e-01 | -8.45e-02 | 1.73e-01 |
| rs10842343 | 12 | 24579079 | A | T | - | - | - | -4.39e-02 | 5.71e-02 | -4.79e-03 | 2.22e-02 | 1.09e-01 | 5.61e-02 | -3.22e-02 | 2.78e-02 | 1.10e-01 | 7.41e-02 | 5.74e-02 | 4.50e-02 |
| rs7971408 | 12 | 47876942 | T | C | - | - | - | -1.14e-01 | 8.99e-02 | -5.25e-03 | 3.49e-02 | -1.29e-01 | 8.89e-02 | 1.59e-02 | 4.36e-02 | -1.15e-01 | 1.18e-01 | -5.18e-02 | 7.17e-02 |
| rs1054442 | 12 | 49389320 | A | C | - | - | - | 7.95e-02 | 5.96e-02 | -9.81e-03 | 2.20e-02 | -5.35e-02 | 5.82e-02 | -4.57e-02 | 2.76e-02 | -4.25e-02 | 7.42e-02 | 6.48e-02 | 4.48e-02 |
| rs7132908 | 12 | 50263148 | A | G | - | - | - | 2.70e-02 | 5.75e-02 | 2.05e-02 | 2.21e-02 | -4.84e-02 | 5.54e-02 | 3.67e-02 | 2.78e-02 | -7.18e-02 | 7.17e-02 | -3.33e-02 | 4.48e-02 |
| rs1131017 | 12 | 56435929 | C | G | - | - | - | -7.41e-02 | 5.71e-02 | -2.48e-03 | 2.17e-02 | -5.07e-02 | 5.70e-02 | -8.28e-03 | 2.73e-02 | -1.78e-02 | 7.25e-02 | -2.34e-02 | 4.38e-02 |
| rs1148006 | 12 | 75978358 | A | G | - | - | - | -3.06e-02 | 6.68e-02 | 3.34e-02 | 2.50e-02 | -2.25e-02 | 6.49e-02 | 1.85e-02 | 3.15e-02 | 2.78e-02 | 8.40e-02 | 8.10e-02 | 4.97e-02 |
| rs7979001 | 12 | 97506357 | A | G | - | - | - | 2.76e-02 | 5.66e-02 | -1.11e-03 | 2.14e-02 | 1.01e-02 | 5.51e-02 | 1.90e-03 | 2.69e-02 | -7.80e-02 | 7.18e-02 | 3.69e-02 | 4.31e-02 |
| rs3764002 | 12 | 108618630 | T | C | - | - | - | -6.47e-02 | 6.48e-02 | -3.82e-02 | 2.47e-02 | 9.57e-03 | 6.25e-02 | 5.49e-03 | 3.08e-02 | -3.86e-02 | 8.76e-02 | 1.37e-02 | 4.91e-02 |
| rs660549 | 12 | 121300988 | T | C | - | - | - | 9.75e-03 | 5.56e-02 | 4.09e-02 | 2.18e-02 | 9.07e-02 | 5.33e-02 | 5.21e-04 | 2.73e-02 | -3.65e-02 | 7.37e-02 | 6.30e-03 | 4.37e-02 |
| rs9548873 | 13 | 40238492 | T | C | - | - | - | 2.07e-02 | 6.08e-02 | 2.89e-02 | 2.27e-02 | -4.55e-02 | 5.78e-02 | 1.23e-02 | 2.85e-02 | 5.21e-02 | 7.40e-02 | -1.31e-02 | 4.54e-02 |
| rs73187215 | 13 | 42646769 | A | G | - | - | - | 1.15e-01 | 9.81e-02 | 8.50e-03 | 3.72e-02 | 8.36e-02 | 9.87e-02 | 1.07e-02 | 4.72e-02 | 2.66e-01 | 1.27e-01 | 3.34e-02 | 7.59e-02 |
| rs9568123 | 13 | 49475780 | A | G | - | - | - | -7.15e-02 | 7.79e-02 | 2.30e-02 | 2.97e-02 | -7.00e-02 | 7.91e-02 | -3.86e-02 | 3.67e-02 | -1.61e-01 | 9.89e-02 | -3.03e-02 | 5.87e-02 |
| rs4886140 | 13 | 59833519 | A | G | - | - | - | 9.70e-02 | 5.97e-02 | 4.73e-03 | 2.30e-02 | 1.01e-01 | 5.88e-02 | -2.80e-02 | 2.91e-02 | 7.52e-02 | 7.58e-02 | -7.84e-02 | 4.71e-02 |
| rs1925047 | 13 | 74600274 | A | C | - | - | - | 3.08e-02 | 6.18e-02 | -1.28e-06 | 2.30e-02 | 2.57e-02 | 5.96e-02 | 1.25e-03 | 2.90e-02 | 4.15e-02 | 7.79e-02 | 2.97e-02 | 4.62e-02 |
| rs11619721 | 13 | 112082513 | T | G | - | - | - | 9.57e-02 | 9.89e-02 | 4.99e-02 | 3.70e-02 | 1.11e-01 | 9.67e-02 | 2.60e-02 | 4.72e-02 | 1.17e-01 | 1.28e-01 | -7.44e-02 | 7.86e-02 |
| rs74499585 | 13 | 112285043 | A | G | - | rs117873997 | - | 1.47e-01 | 1.09e-01 | 2.84e-02 | 3.83e-02 | 1.23e-01 | 1.16e-01 | -7.31e-02 | 5.01e-02 | 2.38e-01 | 1.42e-01 | -1.62e-02 | 7.83e-02 |
| rs10136330 | 14 | 30514335 | T | C | - | - | - | 6.43e-02 | 1.37e-01 | -8.84e-03 | 5.31e-02 | 5.53e-02 | 1.37e-01 | -5.79e-03 | 6.64e-02 | 4.16e-02 | 1.81e-01 | -1.79e-01 | 1.16e-01 |
| rs10138913 | 14 | 60943106 | T | C | - | - | - | 8.04e-02 | 6.20e-02 | -2.58e-02 | 2.32e-02 | 3.67e-02 | 5.98e-02 | 3.14e-02 | 2.89e-02 | 7.62e-02 | 7.98e-02 | -7.57e-02 | 4.73e-02 |
| rs10143972 | 14 | 93850179 | T | C | - | - | - | -3.32e-02 | 7.34e-02 | 4.03e-02 | 2.76e-02 | -7.81e-02 | 7.13e-02 | 6.24e-03 | 3.44e-02 | 2.02e-02 | 9.27e-02 | -7.23e-02 | 5.36e-02 |
| rs10145469 | 14 | 97769834 | A | C | - | - | - | -9.23e-02 | 1.25e-01 | -1.34e-02 | 4.86e-02 | 8.74e-02 | 1.29e-01 | 2.16e-02 | 6.05e-02 | 7.88e-02 | 1.60e-01 | -5.03e-02 | 9.92e-02 |
| rs941520 | 14 | 99709702 | A | C | - | - | - | 4.19e-02 | 5.57e-02 | 2.43e-02 | 2.15e-02 | 1.65e-02 | 5.42e-02 | -1.31e-02 | 2.71e-02 | 3.55e-02 | 6.97e-02 | 6.99e-02 | 4.34e-02 |
| rs12894936 | 14 | 100846991 | T | C | - | - | - | -6.35e-02 | 6.35e-02 | 9.38e-03 | 2.42e-02 | -3.14e-02 | 6.25e-02 | 3.01e-02 | 3.03e-02 | -5.66e-02 | 8.25e-02 | -4.07e-02 | 4.92e-02 |
| rs7178532 | 15 | 23794517 | A | G | - | - | - | 2.05e-02 | 6.05e-02 | -2.55e-02 | 2.32e-02 | -5.05e-02 | 6.24e-02 | 2.31e-02 | 2.95e-02 | 3.88e-02 | 8.06e-02 | -8.13e-03 | 4.69e-02 |
| rs4778356 | 15 | 24183428 | A | G | - | - | - | 7.99e-02 | 9.30e-02 | 6.60e-03 | 3.10e-02 | 1.25e-01 | 9.04e-02 | -2.97e-02 | 3.87e-02 | 8.02e-02 | 1.11e-01 | -3.57e-02 | 6.15e-02 |
| rs8040272 | 15 | 24824016 | A | G | - | - | - | 1.08e-01 | 8.37e-02 | 1.76e-02 | 3.27e-02 | 4.55e-02 | 8.24e-02 | -8.87e-03 | 4.07e-02 | 1.49e-01 | 1.07e-01 | 1.06e-01 | 6.80e-02 |
| rs34513772 | 15 | 40608820 | T | C | - | - | - | 2.30e-02 | 6.04e-02 | -2.02e-02 | 2.27e-02 | 5.54e-02 | 5.90e-02 | -2.68e-02 | 2.86e-02 | 7.38e-02 | 7.94e-02 | -8.67e-02 | 4.53e-02 |
| rs1435753 | 15 | 47925066 | T | C | - | - | - | 8.97e-02 | 5.85e-02 | 1.11e-02 | 2.24e-02 | 5.40e-02 | 5.59e-02 | 4.91e-03 | 2.82e-02 | 4.50e-02 | 7.46e-02 | 4.10e-02 | 4.54e-02 |
| rs28757192 | 15 | 51507610 | T | C | - | - | - | 5.26e-01 | 1.35e-01 | 1.07e-01 | 5.66e-02 | 4.21e-01 | 1.29e-01 | 4.01e-03 | 7.42e-02 | 2.72e-01 | 1.93e-01 | 8.96e-02 | 1.15e-01 |
| rs11852771 | 15 | 54364552 | A | G | - | - | - | -8.34e-02 | 5.80e-02 | -1.72e-02 | 2.22e-02 | -8.14e-02 | 5.93e-02 | 1.10e-02 | 2.78e-02 | 1.83e-02 | 7.58e-02 | -6.51e-03 | 4.46e-02 |
| rs3743266 | 15 | 60781513 | T | C | - | - | - | 2.29e-03 | 5.92e-02 | 1.88e-02 | 2.30e-02 | -3.76e-02 | 5.81e-02 | 5.34e-03 | 2.88e-02 | 1.29e-01 | 8.09e-02 | 1.18e-02 | 4.61e-02 |
| rs72756954 | 15 | 64537300 | C | G | - | - | - | 1.84e-01 | 1.22e-01 | 4.55e-02 | 4.57e-02 | 6.92e-02 | 1.15e-01 | -3.87e-02 | 5.57e-02 | 4.15e-02 | 1.46e-01 | 7.83e-02 | 9.39e-02 |
| rs10153031 | 15 | 67987293 | T | G | rs3784692 | - | rs3784692 | 1.15e-01 | 5.82e-02 | 2.46e-02 | 2.19e-02 | 4.49e-02 | 5.75e-02 | -2.98e-02 | 2.78e-02 | 1.13e-01 | 7.20e-02 | 8.07e-02 | 4.40e-02 |
| rs5742915 | 15 | 74336633 | T | C | - | - | - | -7.28e-02 | 5.35e-02 | 1.89e-02 | 2.17e-02 | -6.77e-02 | 5.46e-02 | 4.08e-03 | 2.73e-02 | -7.08e-02 | 7.04e-02 | 5.64e-02 | 4.38e-02 |
| rs1971554 | 15 | 83406228 | T | C | - | - | - | 3.36e-02 | 6.18e-02 | 4.49e-03 | 2.46e-02 | 3.59e-02 | 6.31e-02 | 1.60e-02 | 3.09e-02 | -7.38e-02 | 8.20e-02 | 2.39e-02 | 4.94e-02 |
| rs12915845 | 15 | 89042467 | T | C | - | - | - | -1.09e-01 | 5.92e-02 | 1.39e-02 | 2.17e-02 | 6.43e-03 | 5.92e-02 | -3.26e-02 | 2.75e-02 | -1.32e-02 | 7.66e-02 | -2.77e-02 | 4.39e-02 |
| rs758747 | 16 | 3627358 | T | C | - | - | - | 1.23e-02 | 6.32e-02 | 1.08e-02 | 2.40e-02 | -1.20e-02 | 6.22e-02 | 4.85e-02 | 3.00e-02 | -1.01e-01 | 8.60e-02 | -9.20e-02 | 4.95e-02 |
| rs1704528 | 16 | 14388750 | T | C | - | - | - | 5.31e-02 | 6.13e-02 | 3.65e-03 | 2.28e-02 | 2.79e-02 | 6.19e-02 | 3.76e-02 | 2.88e-02 | -8.49e-03 | 7.47e-02 | -1.46e-02 | 4.57e-02 |
| rs153793 | 16 | 15542199 | A | G | - | - | - | -1.63e-02 | 5.59e-02 | -1.09e-02 | 2.16e-02 | -3.83e-02 | 5.70e-02 | 2.47e-03 | 2.71e-02 | -7.36e-02 | 7.39e-02 | -1.01e-01 | 4.34e-02 |
| rs112991346 | 16 | 19967668 | T | C | - | - | - | -3.94e-03 | 8.92e-02 | -9.70e-03 | 3.20e-02 | 3.29e-03 | 8.27e-02 | -2.67e-02 | 3.99e-02 | -6.87e-02 | 1.09e-01 | -7.27e-02 | 6.30e-02 |
| rs113388806 | 16 | 24804954 | A | T | - | - | - | -6.51e-03 | 1.55e-01 | 8.40e-03 | 5.53e-02 | -1.80e-01 | 1.41e-01 | -2.26e-02 | 6.87e-02 | 2.57e-01 | 2.13e-01 | 7.50e-02 | 1.14e-01 |
| rs8051833 | 16 | 29896390 | A | G | - | - | - | -4.88e-02 | 5.81e-02 | 3.67e-02 | 2.25e-02 | -3.91e-02 | 5.59e-02 | 5.29e-03 | 2.84e-02 | -9.71e-02 | 7.29e-02 | 2.37e-02 | 4.53e-02 |
| rs3809624 | 16 | 30102802 | T | C | - | - | - | 4.39e-02 | 6.26e-02 | 7.64e-03 | 2.31e-02 | 4.45e-02 | 6.03e-02 | 1.83e-02 | 2.92e-02 | 4.72e-03 | 7.84e-02 | 1.08e-02 | 4.66e-02 |
| rs143461173 | 16 | 52283158 | A | G | rs12599115 | - | rs12599115 | 5.33e-02 | 6.96e-02 | -1.97e-02 | 2.74e-02 | 8.42e-02 | 6.92e-02 | 6.66e-02 | 3.54e-02 | 8.74e-03 | 8.77e-02 | 2.96e-02 | 5.57e-02 |
| rs9972653 | 16 | 53814363 | T | G | - | - | - | -8.43e-02 | 5.76e-02 | -4.78e-02 | 2.20e-02 | 2.01e-02 | 5.55e-02 | 5.53e-03 | 2.75e-02 | 6.04e-02 | 7.13e-02 | -1.80e-02 | 4.43e-02 |
| rs7359336 | 16 | 69733460 | A | G | - | - | - | 7.59e-02 | 5.65e-02 | 2.23e-02 | 2.19e-02 | -3.24e-02 | 5.63e-02 | 1.77e-02 | 2.76e-02 | 1.29e-01 | 7.04e-02 | 1.00e-02 | 4.41e-02 |
| rs4448948 | 16 | 72569236 | A | T | - | - | - | -4.62e-02 | 1.12e-01 | 2.50e-02 | 4.08e-02 | -9.97e-02 | 1.08e-01 | 9.00e-02 | 5.28e-02 | -5.45e-02 | 1.45e-01 | -5.31e-02 | 7.96e-02 |
| rs112752732 | 17 | 1942577 | C | G | - | - | - | 2.65e-01 | 1.32e-01 | 6.98e-03 | 5.13e-02 | 1.91e-01 | 1.23e-01 | -6.10e-02 | 6.64e-02 | 1.45e-01 | 1.77e-01 | 1.69e-01 | 9.63e-02 |
| rs142643995 | 17 | 2017993 | T | C | - | - | - | 7.52e-02 | 1.61e-01 | -1.43e-01 | 6.69e-02 | 1.29e-02 | 1.67e-01 | -1.27e-01 | 8.34e-02 | 4.19e-01 | 1.92e-01 | -1.43e-01 | 1.34e-01 |
| rs12603280 | 17 | 6034754 | A | G | - | - | - | 1.24e-01 | 6.65e-02 | 1.67e-03 | 2.51e-02 | 7.69e-02 | 6.58e-02 | -3.60e-02 | 3.19e-02 | -4.31e-02 | 8.85e-02 | 8.85e-02 | 4.95e-02 |
| rs55680968 | 17 | 7774047 | A | G | - | - | - | 1.14e-01 | 1.10e-01 | -1.87e-02 | 3.99e-02 | 7.99e-02 | 1.09e-01 | -9.06e-02 | 4.90e-02 | 3.39e-02 | 1.33e-01 | 3.39e-02 | 8.21e-02 |
| rs59246405 | 17 | 43123625 | T | C | - | - | - | 5.69e-02 | 5.99e-02 | -6.57e-04 | 2.18e-02 | 6.53e-02 | 5.71e-02 | -3.30e-02 | 2.75e-02 | 7.83e-02 | 7.44e-02 | -1.19e-01 | 4.44e-02 |
| rs11079810 | 17 | 46227846 | T | C | - | - | - | -5.86e-02 | 9.02e-02 | 5.63e-02 | 3.43e-02 | -5.57e-02 | 8.53e-02 | 2.44e-02 | 4.34e-02 | 4.42e-03 | 1.11e-01 | -1.82e-02 | 7.12e-02 |
| rs9635759 | 17 | 49613785 | A | G | - | - | - | 3.97e-02 | 6.11e-02 | 3.85e-03 | 2.39e-02 | 7.67e-02 | 5.96e-02 | 1.92e-02 | 3.00e-02 | 1.82e-01 | 7.97e-02 | -1.37e-02 | 4.82e-02 |
| rs2787487 | 17 | 53209382 | C | G | - | - | - | -9.88e-03 | 5.57e-02 | 2.26e-02 | 2.22e-02 | -2.02e-02 | 5.53e-02 | -2.08e-02 | 2.78e-02 | 6.57e-02 | 7.31e-02 | 5.07e-02 | 4.48e-02 |
| rs7218751 | 17 | 77796437 | A | G | - | - | - | 3.95e-02 | 7.14e-02 | 2.06e-02 | 2.72e-02 | -7.36e-02 | 7.28e-02 | -4.77e-02 | 3.35e-02 | 1.12e-01 | 9.63e-02 | -7.76e-02 | 5.32e-02 |
| rs59652033 | 17 | 77951023 | T | C | - | - | - | 1.31e-02 | 5.99e-02 | -1.89e-02 | 2.34e-02 | 6.98e-02 | 5.88e-02 | 1.93e-02 | 2.93e-02 | -5.12e-02 | 8.10e-02 | 2.23e-03 | 4.70e-02 |
| rs66508321 | 17 | 78739672 | A | G | - | - | - | 5.10e-03 | 5.95e-02 | 1.93e-02 | 2.28e-02 | -3.19e-02 | 5.89e-02 | -5.01e-02 | 2.91e-02 | -3.65e-02 | 7.64e-02 | 5.54e-02 | 4.57e-02 |
| rs2659007 | 17 | 79217478 | A | G | - | - | - | -5.28e-02 | 5.58e-02 | 4.60e-02 | 2.17e-02 | 5.04e-03 | 5.65e-02 | -2.21e-02 | 2.74e-02 | -2.37e-03 | 7.26e-02 | -2.48e-02 | 4.38e-02 |
| rs12937034 | 17 | 79446015 | A | G | - | - | - | -2.44e-02 | 5.85e-02 | 1.39e-02 | 2.21e-02 | -7.59e-02 | 5.67e-02 | 5.09e-02 | 2.79e-02 | 7.37e-02 | 7.67e-02 | 2.86e-02 | 4.46e-02 |
| rs11873906 | 18 | 3813464 | A | G | - | - | - | -7.78e-02 | 6.08e-02 | -3.21e-02 | 2.37e-02 | -4.74e-02 | 6.16e-02 | 1.60e-02 | 3.02e-02 | 9.00e-02 | 8.03e-02 | 3.55e-02 | 4.85e-02 |
| rs1512238 | 18 | 44748467 | A | G | - | - | - | -3.23e-02 | 6.01e-02 | -4.27e-02 | 2.18e-02 | 3.59e-02 | 5.85e-02 | -6.57e-03 | 2.74e-02 | 5.51e-02 | 7.39e-02 | -9.80e-03 | 4.38e-02 |
| rs7239114 | 18 | 45921214 | A | G | - | - | - | 8.02e-02 | 6.01e-02 | -5.37e-03 | 2.17e-02 | 4.38e-02 | 5.84e-02 | 2.75e-02 | 2.73e-02 | 1.10e-02 | 7.80e-02 | -3.10e-03 | 4.37e-02 |
| rs3746037 | 19 | 1828948 | A | C | - | - | - | 9.87e-02 | 6.81e-02 | -1.32e-03 | 2.63e-02 | -1.17e-02 | 6.71e-02 | -1.93e-03 | 3.30e-02 | -3.09e-02 | 8.54e-02 | 5.17e-02 | 5.37e-02 |
| rs169080 | 19 | 4980864 | T | C | - | - | - | 7.35e-02 | 6.03e-02 | -1.47e-02 | 2.24e-02 | -4.68e-03 | 5.89e-02 | 5.30e-02 | 2.79e-02 | -1.72e-01 | 7.93e-02 | 1.49e-02 | 4.49e-02 |
| rs484353 | 19 | 7891767 | A | G | - | - | - | -4.94e-02 | 5.78e-02 | -1.33e-02 | 2.16e-02 | 4.31e-02 | 5.80e-02 | 4.66e-02 | 2.73e-02 | 6.60e-02 | 7.19e-02 | 4.75e-02 | 4.37e-02 |
| rs4804117 | 19 | 9984509 | T | G | rs5004102 | - | rs5004102 | -3.73e-02 | 5.66e-02 | -1.03e-02 | 2.15e-02 | -4.92e-02 | 5.72e-02 | 3.69e-02 | 2.72e-02 | -1.18e-01 | 7.30e-02 | -1.76e-02 | 4.34e-02 |
| rs10422323 | 19 | 13104027 | A | G | - | - | - | -8.21e-02 | 8.58e-02 | -2.48e-03 | 3.25e-02 | -1.63e-01 | 8.56e-02 | -1.28e-02 | 4.09e-02 | -1.50e-01 | 1.16e-01 | 1.09e-02 | 6.52e-02 |
| rs12460047 | 19 | 18346228 | A | G | - | - | - | -1.41e-01 | 6.77e-02 | 1.43e-05 | 2.41e-02 | -3.51e-03 | 6.38e-02 | 1.06e-03 | 3.02e-02 | -8.62e-02 | 8.56e-02 | -3.28e-02 | 4.88e-02 |
| rs11668587 | 19 | 18829770 | A | G | - | - | - | 6.38e-02 | 5.88e-02 | 2.07e-02 | 2.27e-02 | -7.21e-03 | 5.93e-02 | -2.86e-02 | 2.83e-02 | -5.70e-02 | 7.46e-02 | -2.34e-02 | 4.54e-02 |
| rs56367141 | 19 | 31051857 | A | C | - | - | - | -2.40e-01 | 8.61e-02 | -3.53e-02 | 3.22e-02 | -1.24e-01 | 8.68e-02 | 2.39e-02 | 3.96e-02 | -1.50e-01 | 1.11e-01 | 2.33e-02 | 6.34e-02 |
| rs29941 | 19 | 34309532 | A | G | - | - | - | -3.74e-02 | 6.05e-02 | -4.45e-02 | 2.31e-02 | 3.55e-02 | 5.86e-02 | -2.11e-02 | 2.90e-02 | -1.16e-01 | 7.93e-02 | -8.11e-02 | 4.68e-02 |
| rs4804025 | 19 | 47609223 | A | G | - | - | - | -2.31e-02 | 5.73e-02 | -5.89e-04 | 2.37e-02 | -2.91e-02 | 5.80e-02 | -2.97e-02 | 2.97e-02 | -3.21e-02 | 7.48e-02 | 2.93e-02 | 4.80e-02 |
| rs2548458 | 19 | 49209325 | T | C | rs2548459 | - | rs2548459 | 4.69e-02 | 5.72e-02 | 2.22e-02 | 2.18e-02 | 4.06e-02 | 5.56e-02 | 5.42e-03 | 2.74e-02 | 1.85e-02 | 7.20e-02 | 5.78e-02 | 4.41e-02 |
| rs4801809 | 19 | 50334895 | T | C | - | - | - | -8.00e-02 | 1.01e-01 | -3.72e-02 | 4.05e-02 | 2.78e-02 | 9.54e-02 | -4.59e-02 | 5.10e-02 | -9.91e-02 | 1.31e-01 | 2.77e-02 | 7.96e-02 |
| rs2889128 | 19 | 58973929 | A | C | - | - | - | 5.93e-02 | 5.58e-02 | -1.46e-02 | 2.16e-02 | 6.34e-02 | 5.50e-02 | -3.11e-02 | 2.72e-02 | 5.17e-02 | 7.00e-02 | 7.17e-02 | 4.33e-02 |
| rs852061 | 20 | 17109159 | A | C | - | - | - | 2.41e-02 | 6.14e-02 | 4.37e-03 | 2.23e-02 | 3.78e-02 | 5.93e-02 | 3.65e-02 | 2.80e-02 | 1.97e-02 | 7.70e-02 | 7.87e-02 | 4.45e-02 |
| rs1535252 | 20 | 19682834 | T | C | - | - | - | 1.63e-02 | 5.53e-02 | -1.41e-02 | 2.17e-02 | 2.81e-02 | 5.49e-02 | 5.18e-03 | 2.73e-02 | -1.59e-02 | 7.20e-02 | 1.11e-02 | 4.36e-02 |
| rs111558392 | 20 | 20348962 | T | C | rs6046818 | - | rs6046818 | 8.63e-02 | 8.30e-02 | -2.35e-02 | 3.04e-02 | 1.06e-01 | 8.51e-02 | -3.61e-02 | 3.84e-02 | 1.28e-01 | 1.07e-01 | 6.42e-02 | 5.95e-02 |
| rs4813429 | 20 | 21485806 | T | C | - | - | - | 7.06e-02 | 7.39e-02 | -1.05e-02 | 2.84e-02 | -1.86e-02 | 6.96e-02 | 4.87e-02 | 3.64e-02 | 1.28e-01 | 9.41e-02 | 5.42e-02 | 5.85e-02 |
| rs1737894 | 20 | 31054702 | C | G | - | - | - | -6.75e-02 | 5.76e-02 | 4.59e-02 | 2.23e-02 | 1.18e-02 | 5.81e-02 | 3.53e-03 | 2.80e-02 | 3.40e-02 | 7.66e-02 | -1.96e-02 | 4.47e-02 |
| rs2295094 | 20 | 33447915 | A | G | - | - | - | -1.20e-01 | 7.99e-02 | -2.47e-02 | 2.84e-02 | -9.16e-02 | 7.85e-02 | -3.80e-02 | 3.59e-02 | -8.33e-02 | 1.04e-01 | -1.90e-02 | 5.71e-02 |
| rs36093651 | 20 | 37287102 | T | C | - | - | - | -8.08e-02 | 7.19e-02 | -2.26e-02 | 2.56e-02 | 1.10e-02 | 6.84e-02 | 8.94e-03 | 3.19e-02 | 2.80e-02 | 8.71e-02 | 6.99e-03 | 5.12e-02 |
| rs2425674 | 20 | 43529461 | C | G | - | - | - | 7.30e-02 | 5.80e-02 | -7.46e-03 | 2.16e-02 | 4.65e-02 | 5.71e-02 | 1.26e-02 | 2.72e-02 | 4.56e-02 | 7.45e-02 | 1.11e-02 | 4.35e-02 |
| rs3746619 | 20 | 54823805 | A | C | - | - | - | 2.85e-04 | 1.02e-01 | 6.43e-02 | 3.66e-02 | 8.81e-02 | 9.80e-02 | 6.09e-03 | 4.70e-02 | -1.45e-02 | 1.34e-01 | -1.61e-02 | 7.61e-02 |
| rs13043968 | 20 | 54830983 | A | C | - | - | - | -5.87e-03 | 8.71e-02 | 3.90e-02 | 3.48e-02 | -1.00e-01 | 8.42e-02 | 2.51e-02 | 4.39e-02 | 7.77e-02 | 1.06e-01 | 8.54e-03 | 7.10e-02 |
| rs443252 | 20 | 62799680 | T | C | - | - | - | 8.23e-02 | 1.36e-01 | 1.28e-02 | 4.82e-02 | 4.86e-02 | 1.38e-01 | 4.73e-02 | 5.97e-02 | 4.59e-02 | 1.82e-01 | 2.84e-02 | 9.64e-02 |
| rs62229372 | 21 | 37692507 | T | C | - | - | - | -2.90e-02 | 9.06e-02 | -1.80e-02 | 3.29e-02 | 2.63e-02 | 8.86e-02 | 5.84e-02 | 4.02e-02 | -6.93e-02 | 1.14e-01 | -6.42e-02 | 6.74e-02 |
| rs117143374 | 21 | 40555561 | T | C | - | - | - | 1.00e-01 | 8.98e-02 | -3.56e-02 | 3.07e-02 | 1.06e-01 | 8.68e-02 | -6.63e-02 | 3.83e-02 | 3.29e-02 | 1.07e-01 | 7.69e-02 | 6.44e-02 |
| rs151680 | 22 | 22273242 | T | C | - | - | - | 1.37e-03 | 5.42e-02 | 2.76e-02 | 2.15e-02 | 1.76e-02 | 5.41e-02 | 6.90e-03 | 2.71e-02 | 6.95e-02 | 7.36e-02 | -2.60e-02 | 4.33e-02 |
| rs5753377 | 22 | 31293700 | A | G | - | - | - | -6.89e-02 | 6.23e-02 | -1.15e-02 | 2.41e-02 | -1.85e-02 | 6.07e-02 | -7.26e-02 | 3.08e-02 | -1.36e-01 | 8.00e-02 | 3.66e-03 | 4.83e-02 |
| rs4303811 | 22 | 39157755 | A | G | - | - | - | 9.66e-02 | 8.33e-02 | -2.34e-02 | 3.36e-02 | -1.07e-02 | 8.52e-02 | 2.80e-02 | 4.15e-02 | -1.18e-01 | 1.07e-01 | -1.65e-01 | 7.16e-02 |
| rs9614460 | 22 | 45745229 | T | G | - | - | - | 2.74e-03 | 5.84e-02 | 1.92e-02 | 2.30e-02 | -1.08e-02 | 5.78e-02 | 6.21e-02 | 2.92e-02 | -3.63e-03 | 7.65e-02 | 3.48e-02 | 4.65e-02 |
| rs8136272 | 22 | 49678782 | A | T | - | - | - | -1.28e-01 | 6.05e-02 | -2.18e-02 | 2.43e-02 | -1.07e-01 | 6.02e-02 | 5.39e-02 | 3.12e-02 | -7.32e-02 | 7.98e-02 | -1.29e-02 | 4.91e-02 |

Abbreviations: Chr, Chromosome; EA, effect allele; EPIC-CVD, European Prospective Investigation into Cancer and Nutrition – Cardiovascular Disease; HCE, Human Core Exome; OA, other (non-effect) allele; OEE, Omni Exome Express; SE, standard error.

Table S4. Association between age at menarche and risk of cardiovascular disease in the meta-analysis, EPIC-CVD, and the UK Biobank.

|  | **Meta-analysis** | | **EPIC-CVD** | | **UK Biobank** | |
| --- | --- | --- | --- | --- | --- | --- |
| **Outcome/ Age at menarche (year)** | **No. of cases*** | **HR (95% CI)** | **No. of cases*** | **HR (95% CI)** | **No. of cases*** | **HR (95% CI)** |
| **Myocardial infarction** | |  |  |  |  |  |
| <12 | 1,623 | 1.19 (1.06-1.34) | 552 | 1.29 (1.13-1.48) | 1,071 | 1.12 (1.05-1.19) |
| 12 | 1,537 | 1.08 (1.00-1.17) | 666 | 1.13 (1.01-1.26) | 871 | 1.04 (0.97-1.12) |
| 13 | 1,861 | 1.00 (0.94-1.07) | 821 | 1.00 (0.90-1.11) | 1,040 | 1.00 (0.94-1.06) |
| 14 | 1,766 | 1.00 (0.89-1.12) | 847 | 0.96 (0.87-1.06) | 919 | 1.03 (0.97-1.10) |
| 15 | 997 | 1.03 (0.96-1.11) | 485 | 1.06 (0.93-1.21) | 512 | 1.01 (0.92-1.10) |
| ≥16 | 684 | 1.22 (1.11-1.34) | 365 | 1.22 (1.04-1.43) | 319 | 1.22 (1.09-1.37) |
| **Ischaemic stroke** |  |  |  |  |  |  |
| <12 | 974 | 1.20 (1.08-1.33) | 300 | 1.27 (1.08-1.48) | 674 | 1.15 (1.06-1.24) |
| 12 | 974 | 1.07 (1.00-1.15) | 427 | 1.10 (0.97-1.24) | 547 | 1.05 (0.97-1.15) |
| 13 | 1,211 | 1.00 (0.93-1.07) | 569 | 1.00 (0.90-1.11) | 642 | 1.00 (0.92-1.08) |
| 14 | 1,159 | 0.97 (0.86-1.09) | 599 | 0.93 (0.83-1.04) | 560 | 1.01 (0.93-1.10) |
| 15 | 702 | 1.04 (0.87-1.24) | 346 | 0.95 (0.82-1.10) | 356 | 1.12 (1.00-1.24) |
| ≥16 | 481 | 1.21 (1.06-1.39) | 301 | 1.28 (1.08-1.51) | 180 | 1.15 (0.99-1.34) |
| **Haemorrhagic stroke** | |  |  |  |  |  |
| <12 | 299 | 1.02 (0.88-1.20) | 83 | 1.11 (0.87-1.40) | 216 | 0.96 (0.84-1.10) |
| 12 | 316 | 0.98 (0.85-1.13) | 119 | 1.05 (0.87-1.27) | 197 | 0.92 (0.80-1.06) |
| 13 | 448 | 1.00 (0.90-1.11) | 165 | 1.00 (0.85-1.18) | 283 | 1.00 (0.89-1.13) |
| 14 | 424 | 1.03 (0.93-1.15) | 179 | 1.03 (0.87-1.22) | 245 | 1.04 (0.91-1.18) |
| 15 | 251 | 1.13 (0.96-1.32) | 97 | 1.10 (0.88-1.37) | 154 | 1.15 (0.98-1.35) |
| ≥16 | 149 | 1.13 (0.78-1.64) | 82 | 1.39 (1.09-1.78) | 67 | 0.92 (0.71-1.19) |

*Number of cases were taken from the first imputed dataset. The models were adjusted for age, education (high, medium versus low), smoking status (current, ex versus never), and body mass index (kg/m^2^). For EPIC-CVD, the model was stratified by country. Results are presented based on quasi variances using 13 years at menarche as reference category. Abbreviations: CI, confidence interval; EPIC-CVD, European Prospective Investigation into Cancer and Nutrition – Cardiovascular Disease; HR, hazard ratio.

**Table S5. Sensitivity analysis on the association between age at menarche and risk of cardiovascular disease in the meta-analysis of EPIC-CVD and the UK Biobank restricted to women who were included in the Mendelian Randomisation study.**

| **Outcome/Age at menarche (year)** | **No. of cases*** | **HR (95% CI)** |
| --- | --- | --- |
| **Myocardial infarction** |  |  |
| <12 | 1,407 | 1.22 (1.07-1.38) |
| 12 | 1,330 | 1.12 (1.01-1.24) |
| 13 | 1,567 | 1.00 (0.93-1.07) |
| 14 | 1,510 | 1.01 (0.91-1.12) |
| 15 | 865 | 1.05 (0.96-1.14) |
| ≥16 | 571 | 1.23 (1.10-1.37) |
| **Ischaemic stroke** |  |  |
| <12 | 861 | 1.20 (1.06-1.36) |
| 12 | 834 | 1.05 (0.98-1.13) |
| 13 | 1,064 | 1.00 (0.94-1.07) |
| 14 | 1,007 | 0.95 (0.86-1.06) |
| 15 | 601 | 1.02 (0.87-1.21) |
| ≥16 | 416 | 1.22 (1.04-1.43) |
| **Haemorrhagic stroke** |  |  |
| <12 | 260 | 1.00 (0.84-1.18) |
| 12 | 280 | 0.98 (0.85-1.13) |
| 13 | 390 | 1.00 (0.88-1.14) |
| 14 | 367 | 1.03 (0.93-1.14) |
| 15 | 223 | 1.13 (0.99-1.30) |
| ≥16 | 122 | 1.11 (0.76-1.64) |

*Number of cases were taken from the first imputed dataset. The model was adjusted for age, education (high, medium versus low), smoking status (current, ex versus never), and body mass index (kg/m^2^). For EPIC-CVD, the model was stratified by country. Results are presented based on quasi variances using 13 years at menarche as reference category. Abbreviations: CI, confidence interval; EPIC-CVD, European Prospective Investigation into Cancer and Nutrition – Cardiovascular Disease; HR, hazard ratio.

Table S6. Distribution of participant characteristics across thirds of the genetic risk score for age at menarche.

| **Characteristic** | **EPIC-CVD^a^** | | | **UK Biobank** | | |
| --- | --- | --- | --- | --- | --- | --- |
|  | **Lower third of GRS^b^ (n=2,306)** | **Middle third of GRS^c^ (n=2,414)** | **Upper third of GRS^d^ (n=2,650)** | **Lower third of GRS^b^ (n=83,688)** | **Middle third of GRS^c^ (n=83,380)** | **Upper third of GRS^d^ (n=82,911)** |
| Age, years | 52.3 [45.8-59.0] | 52.5 [46.3-59.0] | 52.5 [46.4-59.7] | 57.0 [50.0-63.0] | 57.0 [50.0-63.0] | 57.0 [50.0-63.0] |
| Hypertension | 815 (35.5%) | 861 (35.9%) | 887 (33.8%) | 37,364 (44.7%) | 36,255 (43.5%) | 35,684 (43.1%) |
| Diabetes mellitus | 56 (2.6%) | 60 (2.7%) | 63 (2.6%) | 3,298 (3.9%) | 2,886 (3.5%) | 2,678 (3.2%) |
| Body mass index, kg/m^2^ | 25.5 [22.9-28.6] | 25.0 [22.7-28.4] | 24.9 [22.6-28.1] | 26.3 [23.6-30.1] | 26.1 [23.4-29.6] | 25.8 [23.2-29.3] |
| Height, cm | 161.0 [156.2-165.5] | 161.6 [157.0-166.0] | 162.0 [157.2-166.2] | 162.0 [158.0-166.0] | 162.0 [158.0-167.0] | 163.0 [159.0-167.0] |
| Smoking status |  |  |  |  |  |  |
| Never | 1,262 (54.8%) | 1,357 (56.4%) | 1,414 (53.5%) | 49,940 (59.9%) | 49,695 (59.8%) | 49,063 (59.4%) |
| Ex | 487 (21.2%) | 491 (20.4%) | 608 (23.0%) | 26,015 (31.2%) | 26,120 (31.4%) | 26,348 (31.9%) |
| Current | 553 (24.0%) | 558 (23.2%) | 621 (23.5%) | 7,462 (8.9%) | 7,278 (8.8%) | 7,192 (8.7%) |
| Education |  |  |  |  |  |  |
| Low | 1,001 (44.1%) | 1,031 (43.1%) | 1,139 (43.5%) | 13,803 (16.7%) | 13,670 (16.6%) | 13,593 (16.6%) |
| Medium | 312 (13.7%) | 320 (13.4%) | 348 (13.3%) | 21,062 (25.4%) | 21,078 (25.5%) | 20,877 (25.4%) |
| High | 959 (42.2%) | 1,039 (43.5%) | 1,133 (43.2%) | 47,926 (57.9%) | 47,764 (57.9%) | 47,606 (58.0%) |
| Total cholesterol, mmol/L | 5.9 [5.2-6.6] | 5.8 [5.1-6.7] | 5.9 [5.2-6.6] | 5.8 [5.1-6.6] | 5.8 [5.1-6.6] | 5.8 [5.1-6.6] |
| C-reactive protein, mg/L | 1.2 [0.6-2.6] | 1.1 [0.6-2.5] | 1.1 [0.5-2.4] | 1.4 [0.6-3.0] | 1.4 [0.6-2.9] | 1.4 [0.6-2.9] |
| Ever use of OCP | 1,276 (55.5%) | 1,394 (58.0%) | 1,478 (55.9%) | 67,819 (81.2%) | 67,633 (81.3%) | 67,767 (81.9%) |
| Postmenopausal | 1,304 (56.5%) | 1,387 (57.5%) | 1,521 (57.4%) | 60,997 (72.9%) | 60,744 (72.9%) | 60,251 (72.7%) |
| Age at menopause, years | 49.0 [45.0-52.0] | 49.0 [44.0-51.0] | 49.0 [45.0-52.0] | 50.0 [45.0-52.0] | 50.0 [45.0-52.0] | 50.0 [45.0-52.0] |

Numbers are presented as median [interquartile range] and n (%), as appropriate. ^a^Including data from the EPIC-CVD sub-cohort, ^b^The lower third of the GRS ranged from -3.26 to -1.68. ^c^The middle third of the GRS ranged from >-1.68 to -1.34. ^d^The upper third of the GRS ranged from >-1.34 to 0.253. Abbreviations: EPIC-CVD, European Prospective Investigation into Cancer and Nutrition – Cardiovascular Disease; OCP, oral contraceptive pill; GRS, genetic risk score.

# Supplementary figures

Figure S1. Selection of SNPs.


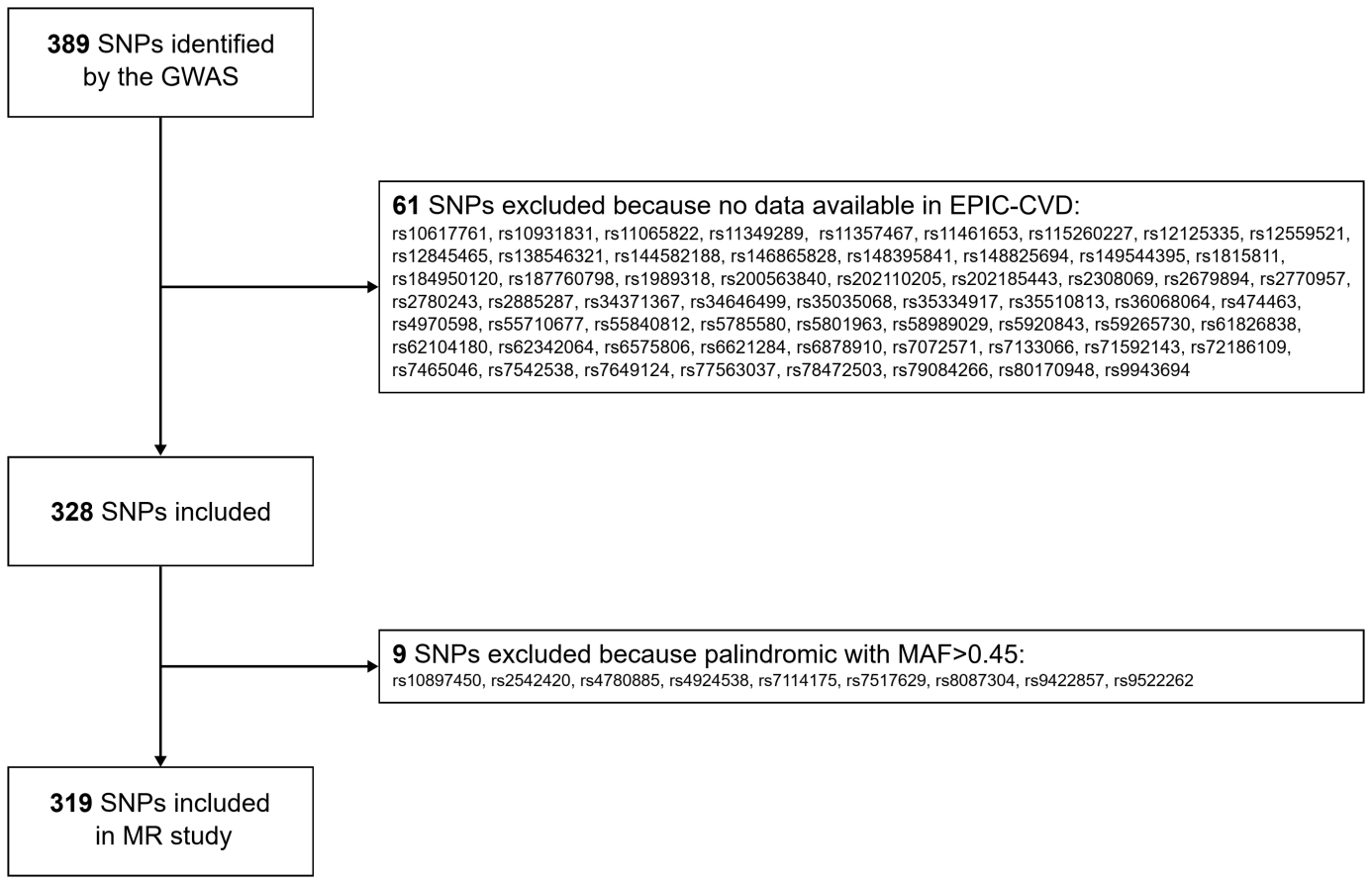


Abbreviations: EPIC-CVD, European Prospective Investigation into Cancer and Nutrition – Cardiovascular Disease; GWAS, genome-wide association study; MAF, minor allele frequency; MR, Mendelian Randomisation; SNP, single nucleotide polymorphism.

**Figure S2. Relationship between genetically proxied age at menarche and other traits across strata of age at menarche in EPIC-CVD and the UK Biobank.**


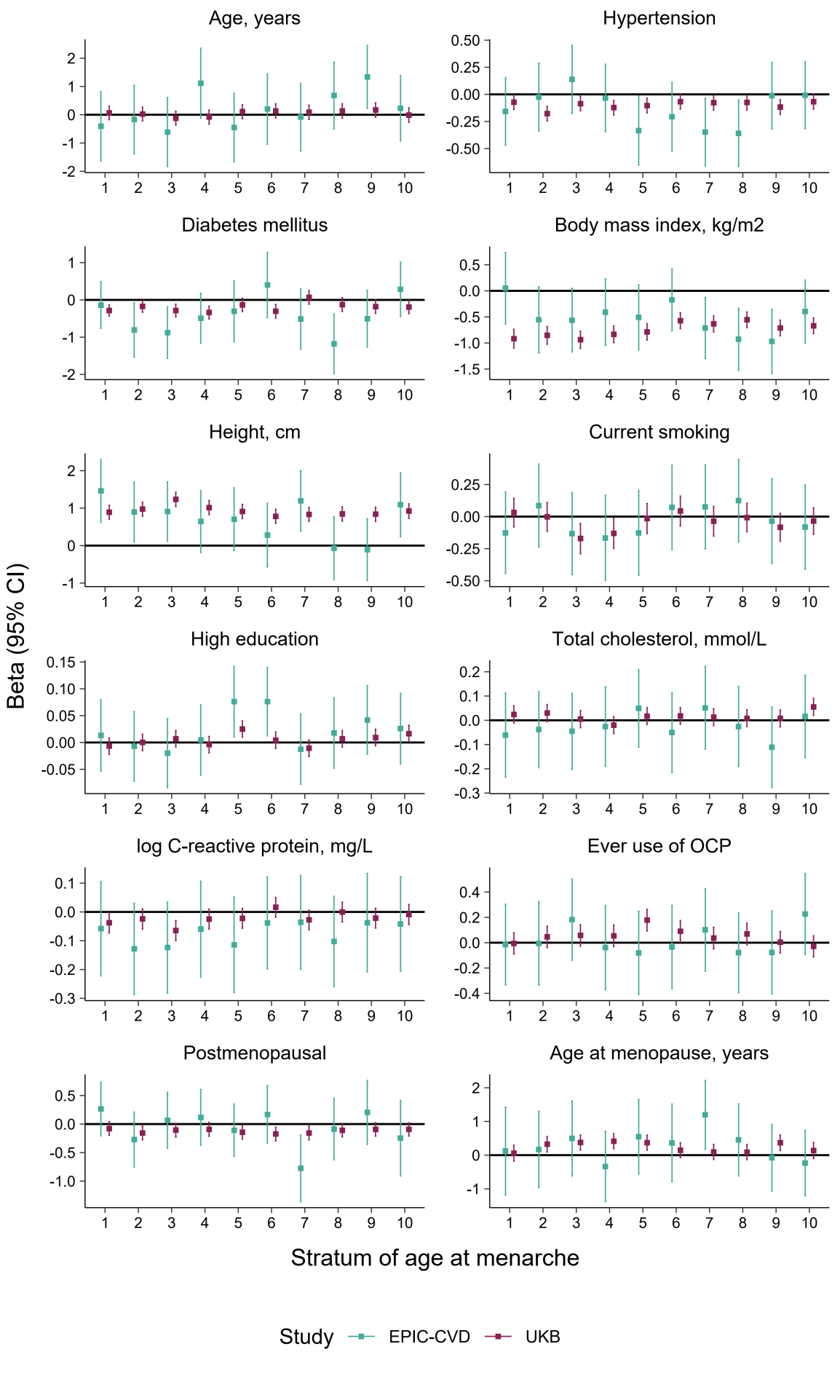


Abbreviations: CI, confidence interval; EPIC-CVD, European Prospective Investigation into Cancer and Nutrition – Cardiovascular Disease; OCP, oral contraceptive pill.

Figure S3. Mendelian Randomisation analysis of genetically proxied age at menarche and risk of myocardial infarction, ischaemic and haemorrhagic stroke using different methods in the meta-analysis of EPIC-CVD and the UK Biobank.


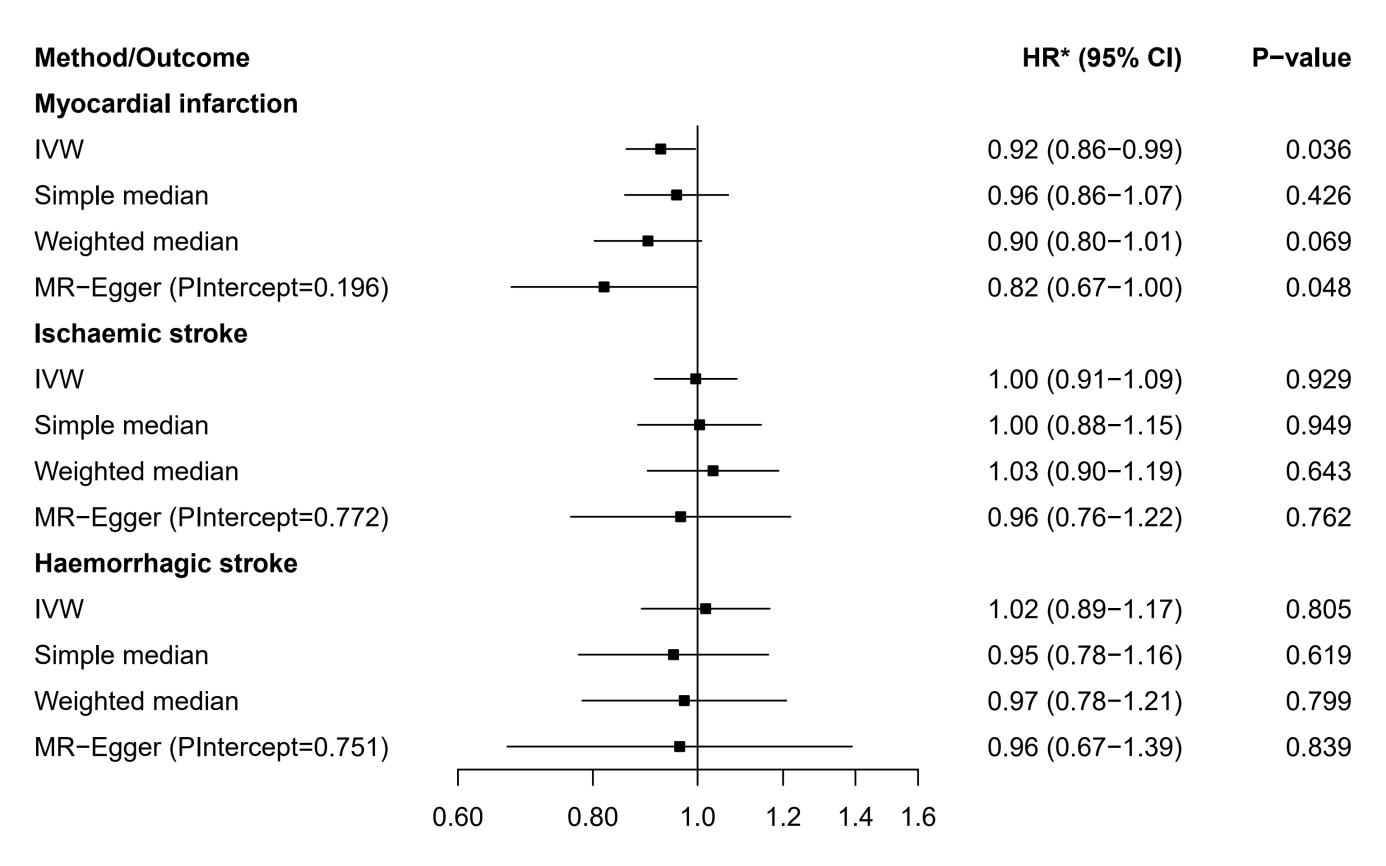


*HR per year older genetically proxied age at menarche. For EPIC-CVD, models were adjusted for age, genotyping array, and the first 10 genetic principal components, and stratified by country. For the UK Biobank, models were adjusted for age, genotyping array, and the first 16 genetic principal components. Abbreviations: CI, confidence interval; HR, hazard ratio; IVW, inverse-variance-weighted.

Figure S4. Mendelian Randomisation analysis of genetically proxied age at menarche and risk of myocardial infarction, ischaemic and haemorrhagic stroke using different methods in EPIC-CVD.


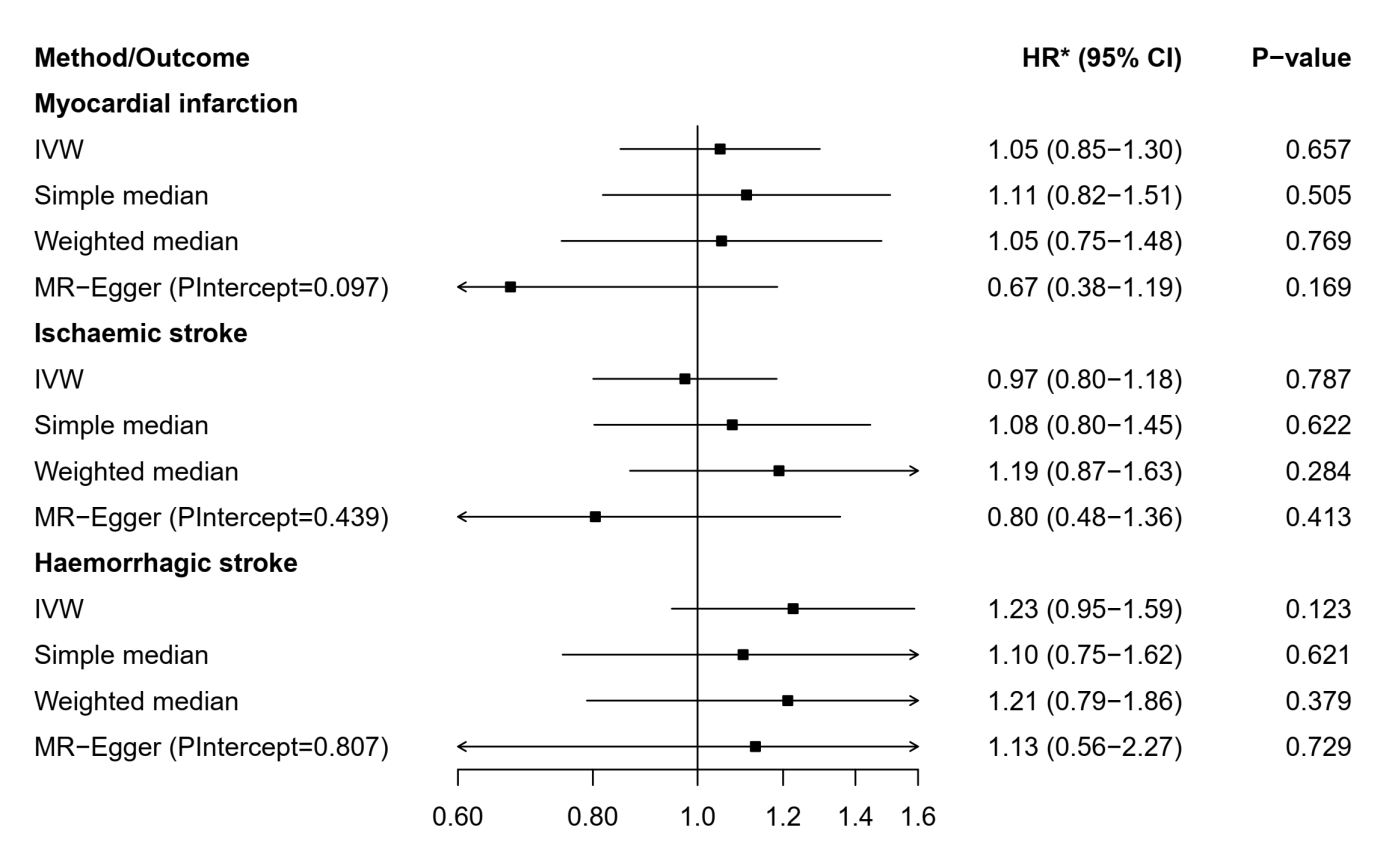


*HR per year older genetically proxied age at menarche. Models were adjusted for age, genotyping array, and the first 10 genetic principal components, and stratified by country. Abbreviations: CI, confidence interval; HR, hazard ratio; IVW, inverse-variance-weighted.

Figure S5. Mendelian Randomisation analysis of genetically proxied age at menarche and risk of myocardial infarction, ischaemic and haemorrhagic stroke using different methods in the UK Biobank.


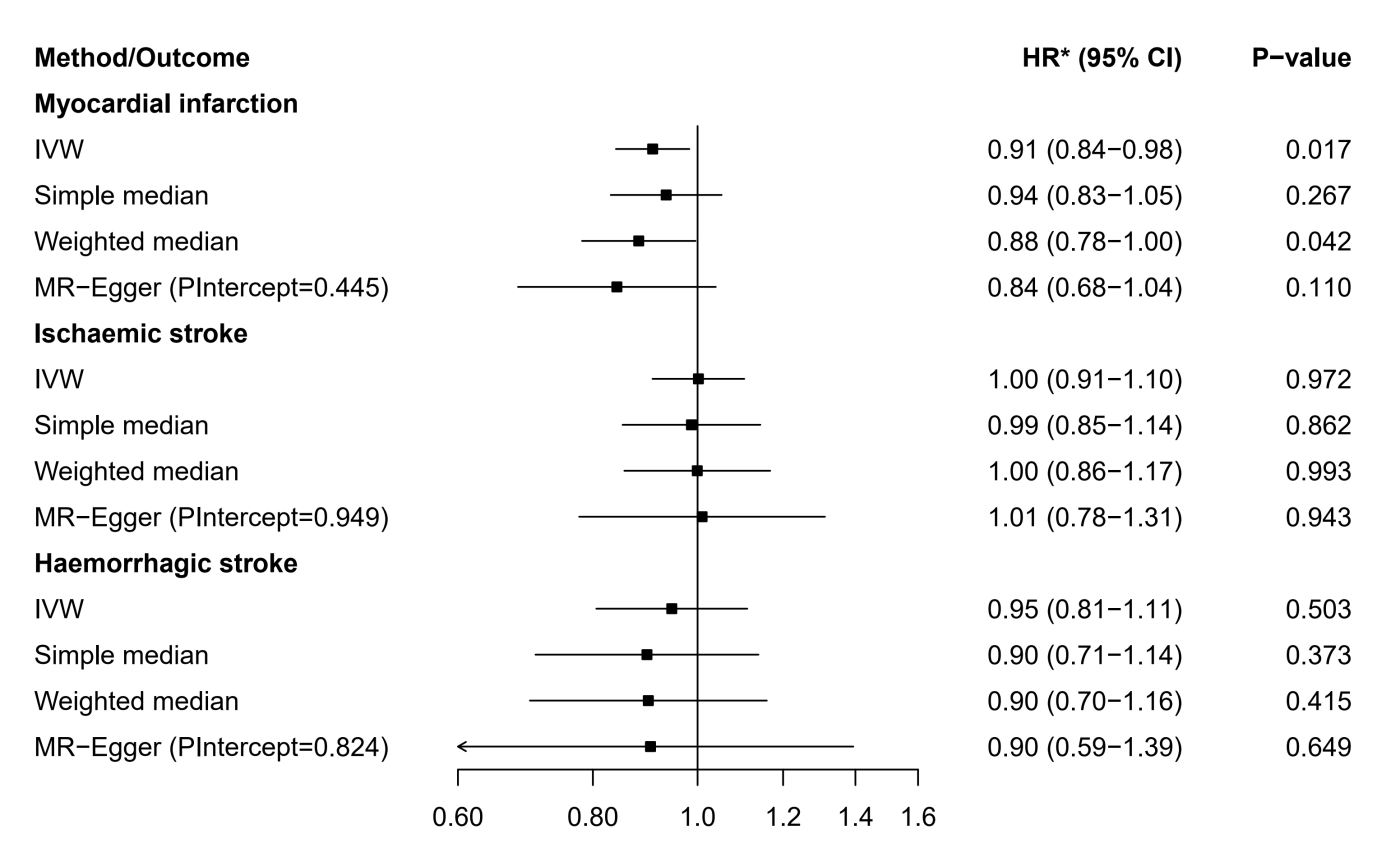


*HR per year older genetically proxied age at menarche. Models were adjusted for age, genotyping array, and the first 16 genetic principal components. Abbreviations: CI, confidence interval; HR, hazard ratio; IVW, inverse-variance-weighted.
